# Cone opponent functional domains in primary visual cortex combine signals for color appearance mechanisms

**DOI:** 10.1038/s41467-022-34020-2

**Published:** 2022-10-25

**Authors:** Peichao Li, Anupam K. Garg, Li A. Zhang, Mohammad S. Rashid, Edward M. Callaway

**Affiliations:** 1 The Salk Institute for Biological Studies, La Jolla, CA 92037 USA; 2 Neurosciences Graduate Program, University of California, San Diego, La Jolla, CA 92093 USA; 3 Medical Scientist Training Program, University of California, San Diego, La Jolla, CA 92093 USA; 4 Present Address: Liangzhu Laboratory, MOE Frontier Science Center for Brain Science and Brain-machine Integration, State Key Laboratory of Brain-machine Intelligence, Zhejiang University, 1369 West Wenyi Road, 311121 Hangzhou, China; 5 Present Address: Wilmer Eye Institute, Johns Hopkins University, 600N Wolfe Street, Baltimore, MD 21287 USA

**Keywords:** Sensory processing, Neuronal physiology

## Abstract

Studies of color perception have led to mechanistic models of how cone-opponent signals from retinal ganglion cells are integrated to generate color appearance. But it is unknown how this hypothesized integration occurs in the brain. Here we show that cone-opponent signals transmitted from retina to primary visual cortex (V1) are integrated through highly organized circuits within V1 to implement the color opponent interactions required for color appearance. Combining intrinsic signal optical imaging (ISI) and 2-photon calcium imaging (2PCI) at single cell resolution, we demonstrate cone-opponent functional domains (COFDs) that combine L/M cone-opponent and S/L + M cone-opponent signals following the rules predicted from psychophysical studies of color perception. These give rise to an orderly organization of hue preferences of the neurons within the COFDs and the generation of hue “pinwheels”. Thus, spatially organized neural circuits mediate an orderly transition from cone-opponency to color appearance that begins in V1.

## Abstract (web summary)

Psychophysical studies predict that retinal cone-opponent mechanisms interact in specific combinations to generate color perception. Here, the authors show that the predicted interactions are implemented by organized circuits in primary visual cortex.

## Introduction

Color vision has intrigued scientists for centuries, largely because it is readily apparent that the colors we perceive are an abstraction from physical reality. For example, the most distant wavelengths of visible light, “red” and “blue” are linked together perceptually through a continuum of extraspectral colors that can only be generated by mixing long and short wavelengths^1^. Among these extraspectral colors is “true red”, a color that human observers report as being neither purplish nor orangish^2^. The percept of true red cannot be generated with monochromatic light, and true red is not a component of the rainbow.

**Fig. 1:**
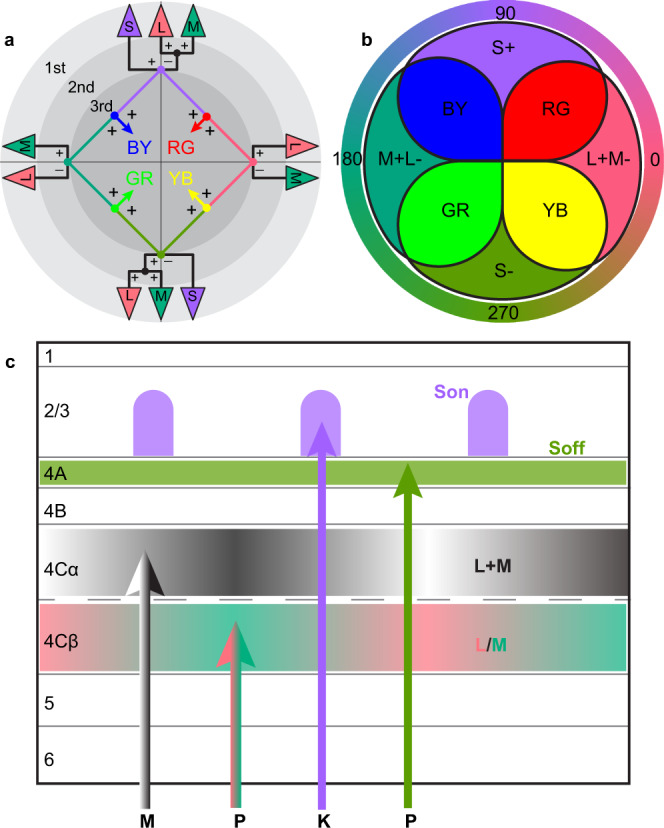
Three-stage zone model of color vision (adapted from Stockman and Brainard^3^) and schematic of M, P, K geniculocortical pathways. **a** At the first stage (outer ring), light is transduced by three types of cone photoreceptors, L (long), M (middle), and S (short) -wavelength sensitive. (Colors in the triangles labeled L, M, or S correspond to the appearance of cone-isolating stimuli that would selectively activate each cone type.) In the second stage, cone signals are integrated through four cone-opponent mechanisms (L+M−, M+L−, S+(L+M)−, and (L+M)+S−) instantiated by retinal circuits connecting specific cone types to retinal ganglion cell types. At the hypothesized third stage, cone-opponent signals are predicted to combine in four specific combinations to generate color-opponent mechanisms, as well as neurons whose activities underlie the perception of four “unique colors” (red—RG; green—GR; yellow—YB; blue—BY). **b** The observed spatial-overlap model of cone-opponent functional domains (COFDs) corresponds to the cone-opponent mixing combinations predicted in the Stage 3 of the Three-stage model. Outer ring illustrates the appearance of colors at different directions in the isoluminant plane of DKL color space. Generation of color preferences in the non-cardinal directions requires mixing of the cone-opponent mechanisms. **c** Anatomical organization of cone-opponent thalamocortical inputs. Cone-opponent signals are transmitted along Parvocellular (P) and Koniocellular (K) pathways from RGCs to different layers in V1 (See Text). Achromatic inputs from the Magnocellular (M) pathway project to layer 4 Cα. The mixing of cone-opponent signals in Stage 3 (**a**) requires neural circuits that span layers 4Cβ, 4 A, and blobs in layer 2/3 to allow mixing between the L/M opponent inputs that target layer 4 Cβ and the S/L+M opponent inputs that target layers 4 A and 3B. Note that the pathways and recipient layers are labeled with different colors corresponding to different cone-opponent signals.

Accordingly, color vision has played a central role in efforts to understand the neural mechanisms that generate perception. Rigorous quantitative psychophysical studies that preceded methods for measuring neuronal activity or deciphering neural connectivity led to a Three-stage model of color perception^3–7^ (Fig. 1a). It was predicted that in the first stage, there are three types of receptors sensitive to long (L), middle (M), and short (S) wavelengths (Fig. 1a, outer ring). We now know that there are, in fact, three cone types (L, M, and S) with the predicted wavelength absorption profiles. The model further predicted a second stage which we now know to be generated by cone-opponent circuit mechanisms in the retina. As illustrated in Fig. 1a, the model predicted mechanisms that would allow color discrimination by comparing activation of L versus M cones (L/M opponency) or by comparing activation of S-cones to the sum of L and M cones (S/L + M opponency). This prediction was later borne out by measurements of the responses of retinal ganglion cells (RGCs) and lateral geniculate nucleus (LGN) neurons that receive direct RGC inputs. We now know that the stage 2 mechanisms are instantiated in parvocellular (P) and “blue-ON” bipolar RGCs, and we understand the retinal circuits that underlie their functional properties^8^. There are five functional types of P cells, four of which are L/M opponents. The P cells are both spatially and color opponent, and the four types of L/M opponent P cells are: L-ON center with M-OFF surround (L + M−); L-OFF center with M-ON surround (L−M + ); M-ON center with L-OFF surround (M + L−); and M-OFF center with L-ON surround (M−L + ). The fifth type of P cell is S-OFF and (L + M)-ON (S−(L + M) + )^9–12^. “Blue-ON” bipolar cells have S-ON and (L + M)-OFF responses (S + (L + M)−) but little or no spatial opponency. Neurons in the LGN have the same cone-opponent properties as their RGC inputs and have laminar spatial distributions consistent with the projections of corresponding RGC types^8^.

Importantly, the cone-opponent mechanisms that account for the ability to discriminate colors do not account for color appearance and color perception. Instead, color appearance involves further interactions between the cone-opponent mechanisms (Stage 3, Fig. 1a). These interactions are expected to give rise to neurons preferring a much larger range of colors than is observed in the retina or LGN but is observed in the primary visual cortex (V1)^13–16^. It is, therefore, apparent that V1 is the locus for mechanisms that begin to generate color appearance, but comparisons of the distributions of neuronal color preferences in V1 versus higher visual areas indicate that further processing in V2, V4, and inferotemporal cortex is likely to be required to establish the populations of neurons that encode for color perception^17–20^.

The Three-stage model predicts specific Stage 3 interactions between cone-opponent mechanisms that are required to generate color opponency and color appearance (Fig. 1a, inner disk; see details below). Other interactions would generate achromatic responses. Because V1 contains both color-selective and achromatic neurons, it is theoretically possible that Stage 3 could be instantiated through random circuits that generate spatially interdigitated achromatic and color-selective neurons. But this scenario is unlikely because previous studies have found that color-selective neurons are spatially organized^21–28^ and because random local interactions between interdigitated achromatic and color-selective neurons would likely degrade color selectivity. Instead, Stage 3 is likely to be implemented through organized circuits. While several observations point to the likelihood that the Stage 3 mechanisms are implemented through organized circuits that generate functional architecture within V1, previous studies have not tested whether known functional domains mediate the required interactions and generate V1 neurons with the predicted color preferences.

Because the Three-stage model preceded the discovery of four types of L/M opponent RGCs, it only considered two L/M opponent mechanisms, L + M− and L−M + , and how they interact with the S + (L + M)− and S−(L + M)+ mechanisms (Fig. 1a). To reconcile this, there must also be precisely organized interactions between the four types of L/M opponent mechanisms. In particular, L + M− should mix with M−L + and L−M + with M + L−, but the remaining combinations should be avoided since they would generate achromatic rather than cone-opponent responses. A recent study demonstrated V1 functional architecture in which neurons with L-ON and M-OFF “red” responses are clustered separately from neurons with M-ON and L-OFF “green” responses^26^, indicating that within these regions, circuits must be organized to mediate the chromatic and not the achromatic interactions. This same study also reported clustering of S+ and S− neurons but did not test color directions that activate both the L/M and S+ or S− mechanisms. Other recent studies have observed functional organization for color preferences but did not use cone-isolating stimuli to identify cone-specific responses and used colors that do not have balanced activation of cone-opponent directions needed to allow quantitative assessment of cone mixing^25,29^.

The predicted cone-opponent mixing combinations of Stage 3 (Fig. 1a, inner disk) are based on explicit models and circuit-level predictions generated by the psychophysics community to account for the appearance of the opponent colors, red and green or blue and yellow^30,31^, as well as “unique colors”^2,3,32,33^. Specifically, the appearance of each of the four “unique colors” (red—RG; green—GR; yellow—YB; blue—BY) requires a particular mixture of the possible combinations between L/M opponent and S + or S- mechanisms, which are L + S+, M + S−, L + S−, and M + S+, respectively. The colors used to illustrate the L-, M-, and S-cones (Fig. 1a) correspond to the appearance of cone-isolating stimuli and are also used to illustrate the cone-isolating color directions (0, 90, 180, or 270°.; Fig. 1b) and LGN afferents (Fig. 1c). The colors in the outer ring of Fig. 1b illustrate the appearance of colors at corresponding directions in Derrington-Krauskopf-Lennie (DKL) color space, which is designed so that the cardinal axes correspond to the cone-isolating directions^34^. The red, yellow, green, and blue regions of Fig. 1b illustrate the perceived colors generated from the mixing of the cone-opponent mechanisms at the indicated locations of overlap.

Mixing between L/M and S/L + M-cone-opponent mechanisms cannot occur before V1 but can potentially be mediated by local circuits within V1. This is because the LGN neurons carrying these different signals project to different layers of V1 (Fig. 1c). L/M opponent signals from LGN project to layer 4 Cβ of V1, while S-OFF LGN afferents terminate in layer 4 A, and S-ON in layer 3B blobs^10^. The most direct substrate for the potential mixing of these opponent mechanisms is layer 4 Cβ spiny stellate neurons that receive L/M opponent inputs and have dense axon projections to layers 4 A and 3B^35,36^. Do these circuits mediate interactions between the cone-opponent mechanisms that follow the Stage 3 mixing rules to generate a functional micro-organization of neurons with systematically organized color preferences (e.g., Fig. 1b)?

Here we present data showing that the predicted combinations for mixing cone-opponent mechanisms to generate color appearance mechanisms are implemented by functionally-organized circuits in the superficial layers of V1. We developed intrinsic signal imaging (ISI) methods to map the locations in V1 that are responsive to the ON and OFF phases of cone-isolating stimuli across large swaths of cortical territory. These maps reveal cone-opponent functional domains (COFDs) where neurons are preferentially activated by the ON or OFF phases of cone-isolating stimuli. Results show that L-ON (L+) and M-OFF (M−) responsive regions are always spatially overlapping, as are L-OFF (L−) and M-ON (M+) regions, to generate L + M− and M + L− domains. The L + M− and M + L− domains occur as adjacent non-overlapping pairs. S+ and S− domains are also organized as non-overlapping pairs. Importantly, the axes joining the L + M−/M + L− pairs are often organized roughly orthogonally (e.g., Fig. 1b) or interdigitated in parallel to the axes joining nearby S+/S− pairs. Quantitative assessment of the overlap of these COFDs shows that they occur in combinations that correspond to the circuits predicted to generate color opponency and unique hues RG, GR, YB, and BY in Stage 3 mechanisms (Fig. 1a, b). The same relationships are observed for the underlying individual neurons assayed with 2-photon calcium imaging (2PCI). To test whether these interactions occur and how they shape the visual responses of individual neurons, we also used visual stimuli that are modulated in non-cardinal color directions in DKL color space^34^. These colors simultaneously activate both L/M and S/(L + M) Stage 2 mechanisms and therefore allow assessment of responses that would require integrated inputs. Assessment of preferred DKL colors with ISI-generated hue-phase maps revealed that interactions at the intersections of COFD pairs form hue “pinwheel” or “linear” structures. Both ISI hue-phase maps and 2PCI of color preferences of individual neurons reveal that the functional architecture and microarchitecture within intersecting COFDs is as expected from relatively linear combinations of cone-opponent signals (Fig. 1b), indicating that circuits in these regions do, in fact, implement the predicted mixing.

## Results

### Overview

**Fig. 2:**
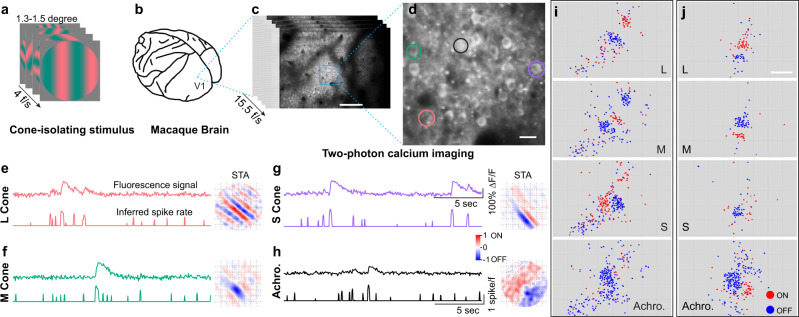
Clustering of neurons with ON/OFF-dominant receptive fields. **a**–**c** Presentation of flashed sine-wave gratings (**a**), L-cone-isolating stimuli (for example) were presented 4 frames per second (4 f/s) to the anesthetized macaque monkey (**b**) during simultaneous two-photon calcium imaging (**c**) in primary visual cortex (V1). Scale bar in **c** is 200 µm. Repetition of experiments is shown in Supplementary Table 1. **d**–**h** Higher magnification view of the imaging region (**d**, scale bar: 20 µm) with four neurons selected as examples to show their fluorescence signals, inferred spike rates, and their receptive fields computed from spike-triggered average (STA) shown in **e**–**h**. The color of traces in **e**–**h** are matched with the color of circles (for indicating neurons only, not the actual ROIs for exacting signals) in **d** to indicate the origin of the signals. Responses of the four different neurons to four different stimuli, L-cone-isolating (**e**), M-cone isolating (**f**), S-cone isolating (**g**), or achromatic (**h**) flashed sine-wave gratings, are shown as four panels. The fluorescence signal (upper trace in each panel) is normalized to the response to blank condition (*∆F/F*). The spike rate (lower trace in each panel) is inferred from raw fluorescence signal. Red colors in the STA images indicate ON subregions of spatial receptive fields, and blue indicates OFF subregions. Each grid in the STA images is 0.2°, and total size is 1.6°. **i**, **j** Functional maps of the locations of neurons with ON-dominant (red dots) and OFF-dominant (blue dots) receptive fields in response to each stimulus type (L-, M-, and S-cone isolating and achromatic (Achro.)). Neurons are organized in ON and OFF clusters. **i**, **j** are two imaging regions from animal A1 (more examples are shown in Supplementary Fig. 1 and also summarized in Supplementary Table 1); both panels illustrate results from two merged imaging planes. Scale bar in **j**: 200 µm; applies to all panels in **i** and **j**.

**Fig. 3:**
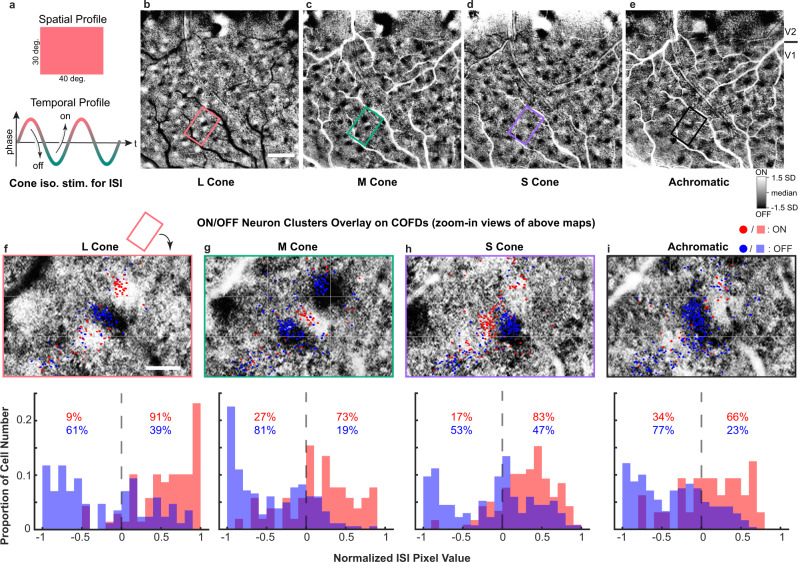
COFDs obtained from ISI are aligned with ON and OFF neuron clusters from 2PCI. **a** Continuous-periodic full-field cone-isolating and achromatic stimuli (top) with corresponding sinusoidal temporal modulation (bottom, L-cone-isolating stimulus is shown). **b**–**e** ISI phase maps reveal ON and OFF functional domains resulting from L-, M-, S-cone-isolating and achromatic stimuli, respectively. Scale bar: 1 mm. (SD: standard deviation of pixel distributions). **f**–**i** ON/OFF neuron clusters aligned with COFD. Panels at top: 2PCI responses of neurons (colored dots, the same as Fig. 2i) to L-, M-, and S-cone-isolating and achromatic stimuli superimposed on corresponding aligned zoom-in ISI phase maps from regions outlined in **b**–**e**. Individual neurons imaged with 2PCI depicted based on whether STA receptive field was predominantly ON (red) or OFF (blue). Histograms at bottom: The ON (red) and OFF (blue) neuron distributions according to the normalized pixel values of aligned ISI phase maps. Positive pixel values represent ON domains of COFDs, negative values represent OFF domains of COFDs. Numbers in the panels are the percentages of ON neurons (red text) or OFF neurons (blue text) in ON versus OFF domains. Scale bar in **f**: 200 µm; applies to **f**–**i**. Note that grids were added in **f**–**i** as landmarks for comparison. The red rectangle above **f** indicates how selected region in **b** is rotated to display **f**; same operation applies to **g**–**i**.

**Fig. 4:**
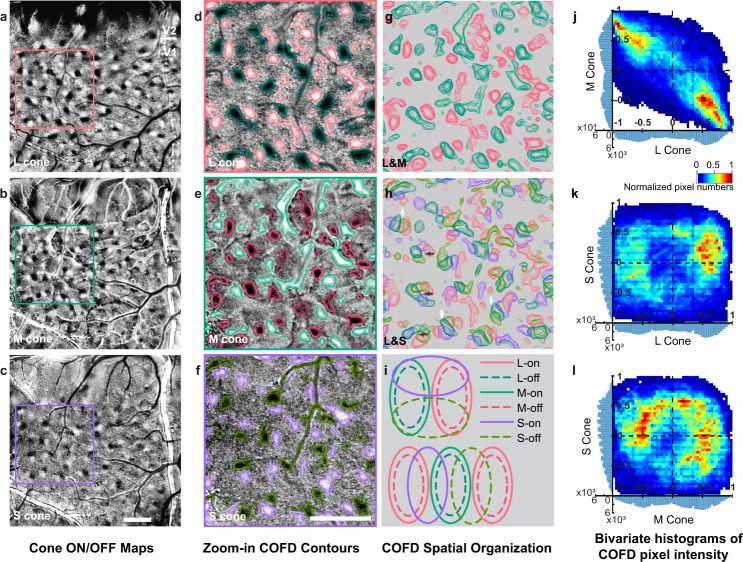
Spatial relationships between L-, M-, and S-COFDs. **a**–**c** COFDs from ISI phase maps in response to full-field periodic L-, M-, and S-cone-isolating stimuli, respectively. Scale bar in **c**: 1 mm; applies to **a**–**c**. **d**–**f** Zoom-in views of maps in **a**–**c**. Color contours for ON and OFF domains are overlaid on images based on ISI pixel values. Scale bar in **f**: 1 mm; applies to **d**–**h**. **g** Merged image of L-COFD contours (**d**) with M-COFD contours (**e**). Same scale as **f**. **h** Merged image of L-COFDs (**d**) with S-COFDs (**f**). Same scale as **f**. Additional examples are shown in Supplementary Fig. 2a. L- and M-COFDs overlap extensively with an anti-phase relationship; L-on COFDs align with M-off COFDs, and L-off align with M-on (**g**, also see **j**). In contrast, S-ON and S-OFF COFDs tend to fall between and partially overlap with L+M− and M+L− COFDs (**h**, also see **k**, **l**). **i** Schematic diagrams of the COFD relationships often seen in **h**. L+M−/M+L− pairs are often grouped with an S+/S-pair such that the pairs have orthogonal axes (**h**, dark arrows, schematized at top of **i**). Alternatively, the pairs are arranged in parallel with S+ and S− COFDs interdigitating between L+M− and M+L− COFDs (**h**, white arrows, schematized at bottom of **i**). **j**–**l** 2D Bivariate histograms illustrating spatial relationships between L, M, and S COFDs. The 1D histograms on the *x-* and *y*-axes display the distribution of normalized pixel values in each category. (Additional cases are shown in Supplementary Fig. 2a). The anti-phase relationships between L and M COFDs are apparent in **j**, while interactions between L or M with S-COFDs are more balanced across all phases (**k** and **l**).

**Fig. 5:**
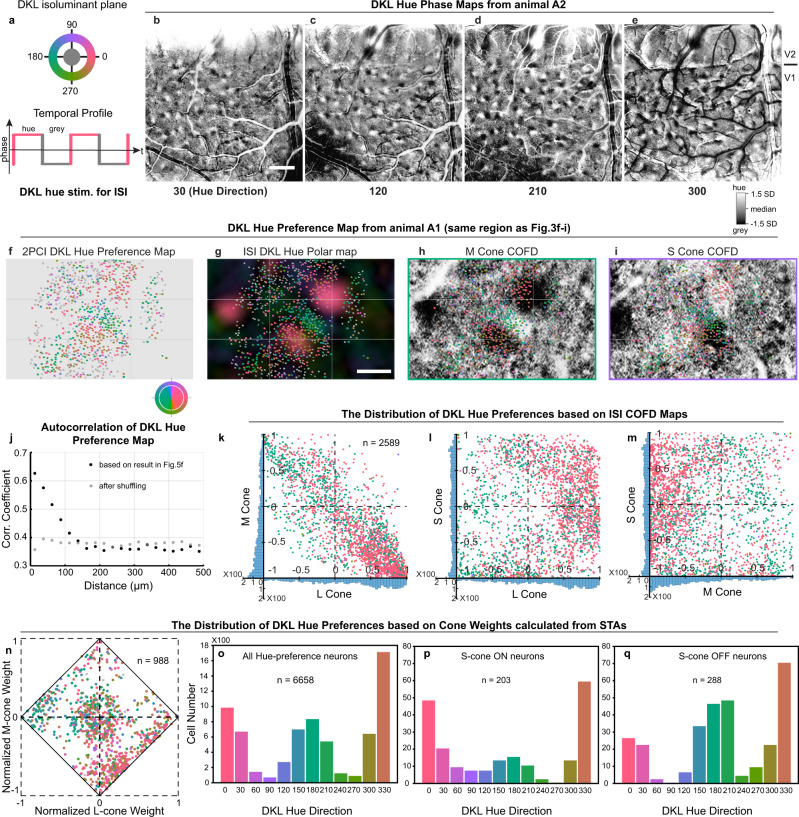
DKL hue map and its relationships to COFDs and Cone weights. **a** Schematic of stimulus presentation. **b**–**e** ISI hue-phase maps in response to 4 hue directions (animal A2). Scale bar in **b**: 1 mm; applies to **b**–**e**. (SD: standard deviation of pixel distributions). **f** DKL hue preference map from same imaging region as Fig. 3f–i. Individual neurons are depicted based on their preferred DKL hue or gray (not hue selective). Color key indicates the relationship between the hues for plotting the map (outer ring) and hues used for stimulation (inner disk). **g** DKL hue preference map (from **f**) overlaid on DKL hue polar map from ISI. **h**, **i** DKL hue preference map (from **f**) overlaid on M-cone and S-cone COFDs (from Fig. 3g, h). Scale bar in **g**: 200 µm; applies to **f**–**i**. **j** Autocorrelation of neuronal hue preferences from **f** as a function of cortical distance. Black dots are from actual data shown in **f**; gray dots are from the same data after shuffling. **k**–**m** Relationships between ISI COFD organization and DKL hue preferences based on individual neurons identified by 2PCI. Each dot represents a neuron which is within COFD, and its *x y* coordinates are the mean normalized pixel values within that neuron from COFD maps (see Methods). Histograms on *x* and *y*-axes show the distributions of neurons based on normalized pixel values from ISI. Individual neurons are colored according to their preferred DKL hue. **n** Relationships between DKL hue preferences and cone weights calculated from STAs. 988 neurons with significant L, M, or S-cone STA from 5 imaging regions (10 planes, see Supplementary Fig. 5) of A1 were selected to calculate normalized cone weights. Normalized L and M-cone weights are plotted on the *x*- and *y-*axes and S-cone weights are implicit as the distance from the edge. Data point colors show preferred DKL hues (or gray for not hue selective). **o**–**q** Distributiond of preferred DKL hues of all hue-selective neurons (**o**), hue-selective neurons with significant S-ON STA (**p**), or with significant S-OFF STA (**q**). (5 imaging regions, 10 planes, from A1.).

**Fig. 6:**
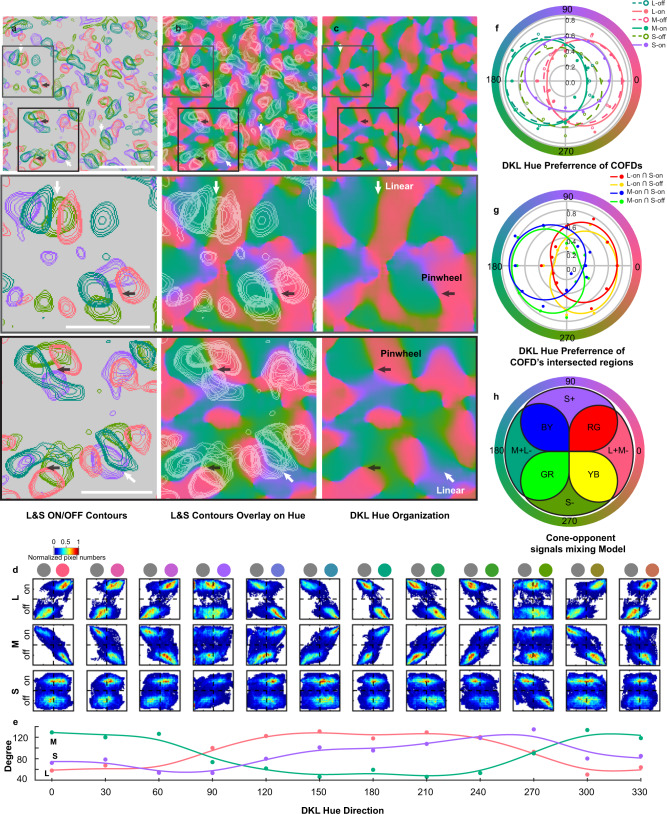
The functional organization of DKL hue-direction map and its relationship with COFDs. **a**–**c** COFDs contours (**a**), DKL hue-direction map (**c**), and spatial overlay (**b**). Second and third row panels are zoom-in views of selected regions. Both pinwheel (black arrows) and linear (white arrows) structures exist in DKL hue-direction map. Scale bars: **a** top 1 mm, **a** middle and bottom 0.5 mm; applies to **a**–**c**. Note that the first image in **a** is same as Fig. 4h. The cortical region in the first row of **a**–**c** is same region shown Fig. 4a–h. **d**, **e** Spatial relationship between DKL hue-direction domains and each type of COFD. Rows in **d** correspond to comparisons of hue maps with L (top), M (middle), and S (bottom) COFDs. DKL hue directions indicated at bottom of **e** also apply to columns in **d** and corresponding colors (and gray) are shown at the top of **d**. The bivariate histograms show systematic relationship between COFDs and DKL hue-direction domains. **e** The angles defined by the top 10% of pixel densities in each bivariate histogram for each cone type plotted in relation to each hue direction. *Y*-axis is the angle calculated from each bivariate histogram. **f** Hue tuning curves calculated from the means of ISI pixel values within each COFD region in response to each DKL hue direction. **g** Hue tuning curves calculated from the means of ISI pixel values within each COFD-intersection region in response to each DKL hue direction. Method of curve fitting used in **f** and **g** is described in Methods. Another case is shown in Supplementary Fig. 4e, f. **h** Cone-opponent signals mixing model based on COFDs. The spatial organization and overlap of COFDs follows Stage 3 mixing rules for color opponency and color appearance mechanisms, and creates hue tuning. The organization allows four intersections between L/M and S/(L+M) cone-opponent mechanisms at the four overlapping regions of COFDs, to generate color appearance mechanisms and encompasses the four “unique colors” (red—RG; green—GR; yellow—YB; blue—BY).

**Fig. 7:**
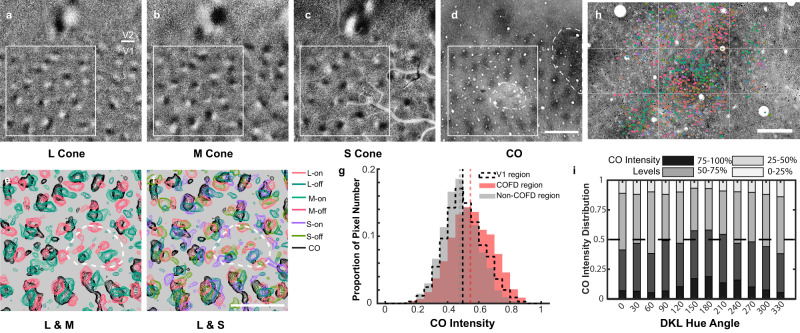
Spatial relationship between COFDs and CO blobs. **a**–**c** L- (**a**), M- (**b**), and S-cone (**c**) COFD maps of V1 and V2 from animal A5. **d** CO image after alignment. It is the same region shown in **a**–**c**. Scale bar in **d**: 1 mm; applies to **a**–**d**. **e**, **f** Zoom-in view of the COFD and CO contours from the region within the white square shown in **a**–**d**. More cases are shown in Supplementary Fig. 7a. Scale bar in **f**: 1 mm; applies to **e** and **f**. **g** Histogram of CO intensity distributions in COFD, non-COFD and whole V1 regions. Dashed lines are median of the distribution in each region. The CO intensity distribution in COFD regions is significantly higher than that in non-COFD regions (*n* = 96 COFD regions, *P* = 0.0062, Wilcoxon signed rank test, two-sided). Note that dotted white outline in **d**–**f** indicates region restricted from quantitative analysis due to damage by an electrode penetration (subsequent to ISI data collection) and loss of CO staining. **h** DKL hue preference map overlays on top of cytochrome-oxidase (CO) histology. Scale bar in **h**: 200 µm. Note that h is the same region shown in Fig. 3f–i and Fig. 5f–i. Grids were added as landmarks for comparison. **i** CO intensity distribution of DKL hue preference neurons. Each column is the CO intensity distribution (grouped as four levels) of pixels corresponding to the neurons (based on the alignment shown in **h**) prefer the hue direction shown on *x*-axis. Results are from 5 regions (10 planes, see Supplementary Fig. 5); **h** is one example.

As introduced above, previous studies have reported clusters of V1 superficial layer neurons that respond to L + M−, L−M+, S+, or S− stimuli^26^ but did not quantitatively assess spatial organization related to the possible overlap of these properties and did not test responses to colors modulated along non-cardinal directions that simultaneously modulate both L/M and S/(L + M) cone-opponent mechanisms. To explicitly investigate possible interactions between cone-opponent mechanisms and whether they occur functionally to generate neurons with preferred colors reflecting those interactions we: (1) developed and validated ISI methods to reveal cone-opponent functional domains (COFDs) across large V1 regions (Figs. 2,3); (2) quantitatively assessed the spatial overlaps between each combination of ISI COFDs (Fig. 4); (3) used stimuli in non-cardinal DKL color directions to produce ISI hue-phase maps (Fig. 5) and hue-direction maps (Fig. 6) demonstrating that interactions between cone-opponent mechanisms produce orderly maps and hue pinwheels; (4) used 2PCI to assess the relationships between color direction tuning of individual neurons and their locations within the COFDs and demonstrate that preferred colors reflect the predicted mixing between cone-opponent mechanisms (Fig. 5). Together these observations demonstrate that V1 circuits generate COFDs where cone-opponent signals are combined to instantiate the Stage 3 color appearance mechanisms. Finally, we quantitatively assessed the relationships between COFDs and cytochrome-oxidase (CO) histology (Fig. 7).

We conducted ISI and 2PCI to map the functional architecture and measure the visual responses of neurons in superficial layers of V1^25^. Here we present data collected from seven adult male and female macaque monkeys (*M. fascicularis*) (see Supplementary Table 1). All data were collected from anesthetized animals following procedures approved by the Salk Institute Institutional Animal Care and Use Committee. We present ISI data from five animals (A1, A2, and A5–A7) and 2PCI data from three animals (A1, A3, and A4). A previous publication^25^ described limited data from 2 of the animals (A3 and A4) subjected to 2PCI; data presented here include responses to visual stimuli and analyses not described in the previous publication.

### Cone-opponent functional domains in V1

We used the 2PCI and ISI imaging methods described above to reveal COFDs in V1. We begin by describing the functional microarchitecture of cone inputs to individual neurons measured with 2PCI imaging. We presented flashed sine-wave gratings (Fig. 2a) while monitoring changes in fluorescence of superficial V1 neurons (150–310 µm depth, Fig. 2b–d) expressing the genetically encoded calcium indicator GCaMP6f (Fig. 2c, d). These sine-wave gratings consisted of 4 sets with different colors, which were L-, M-, or S-cone-isolating or achromatic, and they were presented in separate stimulus blocks. Each set was generated using the same Hartley basis function^37^, therefore, the orientations, spatial frequencies (SFs), and spatial phases were identical between each set. Fluorescence changes were converted to inferred spike-rate time sequences that were then used to calculate spike-triggered averages (STA) of stimuli as linear estimates of receptive fields (Fig. 2e–h). 2PCI from nine imaging regions (16 planes, 1168 × 568 µm or 1100 × 725 µm) yielded data from 13,117 neurons that were visually responsive to drifting gratings. A subset of those visually responsive neurons (2420 of 13,117 neurons, 18.4%) had significant STAs to one or more of the Hartley stimulus sets. These included 1578 neurons with significant STAs to the achromatic stimulus set, 596 to L-cone isolating, 607 to M-cone, and 600 to S-cone. That only a minority of neurons have significant STAs is expected from the prevalence of complex cells in superficial V1 that do not linearly integrate responses to flashed grating stimuli. The smaller numbers of neurons responsive to L and M-cone-isolating versus achromatic stimuli is expected from the low cone-contrasts of the L and M-cone-isolating stimuli (18–18.5%, L and M-cone-contrasts were matched) imposed by the properties of the CRT monitor, versus 98–99% achromatic stimulus contrast. The small minority of neurons with significant S-cone STAs cannot be attributed to the cone contrast of the S-cone stimulus, which was 89.9–90.2%.

As expected from previous studies mapping receptive fields of V1 neurons in response to cone-isolating stimuli^38–40^, receptive field structures varied between stimulus sets and between neurons. We do not further consider the fine spatial structure of receptive fields here. If a neuron had a significant STA to one or more of the flashed sine-wave gratings sets, then we calculated its ON or OFF dominance (see Methods), and plotted the ON/OFF dominance map in red/blue, based on the locations of neurons. Maps from all imaging regions are presented in the main and supplementary Figures. (Figs. 2,3 and Supplementary Fig. 1). Here, we call attention to representative examples from two imaging regions in Fig. 2i, j and Fig. 3f–i. In all imaging regions, neurons with significant STAs occupied a small and relatively contiguous region within the imaging window. This organization is best appreciated in the context of maps showing the locations of all visually responsive neurons, relative to the locations of neurons with significant STAs (compare Fig. 5f–i to Fig. 3f–i). It is apparent from these maps that neurons with dominant responses to the ON versus OFF phases of each stimulus set are clustered together for each of the four stimulus sets. Furthermore, clusters of neurons with L-ON dominance overlap with M-OFF dominance clusters, while L-OFF clusters overlap with M-ON. The smaller numbers of neurons with significant S-cone STAs prevent definitive identification of the spatial relationships of S-ON and S-OFF clusters to the L and M clusters based on this relatively subjective assessment. (We present detailed neuron-by-neuron quantitative comparisons of the signs and magnitudes of cone weights to each cone-isolating stimulus below, Fig. 5n). These cone and achromatic ON/OFF dominance maps suggest that within V1 there are functional architectures where neurons with significant STAs are clustered together, that ON and OFF-dominant neurons of each type are further clustered, and that the organized overlap between L and M-cone clusters creates L + M− and M + L− cone-opponent clusters.

We inferred that this cone-opponent functional architecture might be visualized across much larger V1 regions using ISI, and allow its relationship to cytochrome-oxidase (CO) “blobs” and “interblobs”^41^ to be revealed by aligning postmortem CO histology to ISI maps^25^. To generate ISI ON/OFF-phase maps corresponding to the ON/OFF phases of L-, M-, and S-cone-isolating and achromatic stimuli, we presented full-field stimuli that were temporally modulated between ON and OFF phases of each stimulus type (L, M, S, and achromatic) at 0.017 Hz, 0.08 Hz or 0.1 Hz (Fig. 3a). Subsequent Fourier analysis of the ISI images yielded ON/OFF-phase maps for each stimulus type^42^. In all five animals imaged, and in response to each stimulus type, the phase maps showed clear patches of ON and OFF signals surrounded by regions with no phasic response, extending across the full extent of V1 (Figs. 3, 4, 6, Supplementary Fig. 1, 3b). (Phase maps in the adjacent visual cortical area V2 are not further explored here.) Furthermore, within each patch, there were invariably adjacent pairs of alternating ON and OFF domains. Because of the unknown latency of the hemodynamic signal, correct signs cannot be assigned to the phase maps without additional measurements from another modality with greater temporal sensitivity. We identified the correct phases using electrophysiological recordings (Supplementary Fig. 9) within imaged domains for two of five animals (A2 and A5) and using 2PCI for two of the remaining animals (A1 and A6). For the last animal (A7), similar phase maps were generated (Supplementary Fig. 2b), but we did not identify the phase signs. We assume that the hemodynamic signal latency is consistent between every subregion within V1 for each animal. Alignment of ISI phase maps to the ON/OFF dominance map measured by 2PCI demonstrates a clear correspondence between the two imaging modalities (Fig. 3f–i and Supplementary Fig. 1). We refer to the V1 regions with strong ON and OFF phasic responses to temporally modulated cone-isolating stimuli as cone-opponent functional domains (COFDs). To illustrate and quantify spatial relationships between COFDs, we generated contour maps based on the filtered and normalized pixel values from each phase map (e.g., Fig. 4d–h). The contours shown correspond to the top and bottom 15% values.

### COFD interactions follow Stage 3 cone-opponent mixing rules

Based on their spatial overlaps, the COFDs in V1 appear to follow the cone-opponent mixing rules predicted by the Three-stage model (Fig. 1a). In particular, L-ON functional domains overlap with M-OFF and L-OFF overlap with M-ON, but there is minimal overlap between L-ON and L-OFF or between M-ON and M-OFF (Fig. 4a, b, d, e, and g; see also higher power images in Fig. 6a–c). This overlap generates L + M− and M + L− domains that appear to intersect with both S+ and S− domains (Fig. 4c, f, h). Two different types of intersections appear to be common within these maps. In many cases, an isolated set of COFDs includes one L + M−/M + L− pair and one S+/S− pair, in which the axes connecting the L/M COFDs are perpendicular to the axes connecting the S COFDs (e.g., dark arrows in Fig. 4h, schematic in Fig. 4i, top). Other interactions occur through a parallel arrangement, with S+ and S− domains interdigitated between the L + M− and M + L− domains (e.g., bright arrows in Fig. 4h, schematic in Fig. 4i, bottom). These spatial relationships potentially allow the S+ and S− domains to mix with the L + M− and M + L− domains in the combinations predicted by Three-stage model (Figs. 1a and 4i), suggesting that the COFDs are the substrate for implementation of the cone-opponent signal mixing rules that begin to generate color appearance mechanisms.

To more rigorously test this possibility, we next quantified the relationships between the L, M, and S-COFDs. The COFD map of each cone type was thresholded to generate a mask to select ON- and OFF-phase regions, then masks from all three cone type maps were merged to create a final COFD mask including L, M, and S-COFD regions. Then using normalized pixel values from L, M, and S-COFD maps (range: [−1 to 1]), each pixel within the COFD mask was assigned three values corresponding to the modulation (including magnitude and sign) by each of the three cone-isolating stimuli at that pixel. Pixel-by-pixel comparisons were then made across each of the three possible comparisons of cone types (M to L, S to L, and S to M). Typical results are illustrated in Fig. 4j–l. Similar results were observed for all 4 animals with known ON/OFF phases (Fig. 4 and Supplementary Fig. 2a). It can be seen that for the L-cone versus M-cone comparisons, significantly more pixels (83.69%) fall into the quadrants corresponding to responses of opposite sign (L + M− and M + L−) than in the sign-matched quadrants (L + M+ and L−M−) (*P* = 0.0002, Kolmogorov–Smirnov test, two-sided). In contrast, comparisons between S-cone and either L- or M-cone maps generate pixels relatively evenly distributed across all four quadrants, as predicted. In particular, for the LS comparison, 42.66% of pixels fall into the opposite-sign quadrants (2nd and 4th quadrants) and 57.34% into the same-sign quadrants (1st and 3rd quadrants, *P* = 0.2753, Kolmogorov–Smirnov test, two-sided). And for the MS comparison, 56.48% of pixels fall into the opposite-sign quadrants and 43.52% into the same-sign quadrants (*P* = 0.7710, Kolmogorov–Smirnov test, two-sided). Similar results are observed for 3 additional animals/imaging regions (Supplementary Fig. 2a and legend).

In addition to interactions between cone-opponent mechanisms within a 2-dimensional isoluminant plane (as illustrated in Fig. 1a, b), a full accounting of color space would also include color directions that modulate along the achromatic axis. As described above (Fig. 3i), we also observe ON/OFF-phase maps in response to full-field achromatic stimuli; these overlap extensively with and have a similar structure to COFDs (Fig. 3; Supplementary Fig. 3). To assess whether the achromatic ON/OFF domains might contribute to the generation of a diversity of selectivities for color directions with achromatic modulation, we quantified the relationships between COFDs and achromatic domains, as described above for the relationships between the three COFDs. Pixel-by-pixel comparisons between the achromatic domains and each of the COFDs (L, M, or S) are illustrated in Supplementary Fig. 3d–f. Consistent with a possible contribution of achromatic modulation to color tuning of the underlying neurons, both the ON and OFF achromatic domains overlap extensively with both ON and OFF regions of all three types of COFDs. This contrasts with an alternate hypothesis in which the achromatic domains do not contribute to color tuning and would instead only overlap with COFD pixels of matching sign. Such same-sign overlap would be expected to generate relatively achromatic responses without substantial chromatic modulation.

### DKL hue-direction maps and hue pinwheels

The above observations show that the overlap of the COFDs follows the rules required to integrate cone-opponent signals in the combinations that would generate color opponency, but do not assess functional mapping that might be present to a larger range of intermediate colors predicted from mixing within COFDs. They also do not test whether mixing occurs at the level of individual neurons or how neurons within the overlapping regions respond to a larger range of hues. For example, are the ISI phase responses to colors that concurrently modulate both L/M and S/(L + M) cone-opponent mechanisms predictable from the COFD maps, as expected from the mixing of cone-opponent inputs? Do the individual neurons within the COFDs prefer colors that would be predicted from the mixing of the cone-opponent signals within the COFDs? Answering these questions is central to assessing whether interactions across the COFDs implement the Stage 3 mechanisms.

To address these questions, we selected as visual stimuli a set of 12 hues spaced evenly in the isoluminant plane of DKL color space^34^. We chose the DKL isoluminant plane because there is a close correspondence between DKL hues and the cone-opponent signals^13^, allowing straightforward predictions of hue preferences expected from linear integration of cone-opponent inputs. The 12 DKL hues were presented as drifting gratings for 2PCI or temporally modulated full-field stimuli for ISI, and the responses of neurons within the COFDs were imaged. We generated both ISI hue-phase maps (Fig. 5b–e and Supplementary Fig. 4a), and 2PCI hue maps (Fig. 5f, g and Supplementary Fig. 5) to reveal the overall functional architecture and the microarchitecture of hue preferences of individual neurons. Within the DKL isoluminant plane, hue directions of 0 and 180° correspond to the L + M− and M + L− directions and appear pinkish and greenish, respectively, while 90 and 270° correspond to S+ and S− and appear violet and lime (Fig. 5a and Supplementary Fig. 8b). Intermediate hues are predicted from the mixing of the cone-opponent signals that modulate along these cardinal axes. The L-, M-, and S-cone increments were matched for the DKL stimuli presented to animal A1 (both ISI and 2PCI experiments), while for animal A2 (ISI experiments only), all cone increments were set to the maximum cone-contrasts achievable with the monitor, substantially increasing the S-cone-contrasts relative to L and M-cone-contrasts (Supplementary Fig. 8b). These differences are noted where relevant, below.

Similar to ISI phase maps generated with cone-isolating stimuli (e.g., COFDs), the hue-phase maps generated using DKL stimuli were composed of ON/OFF pairs with strong signals surrounded by regions with weak or no phasic modulation (Fig. 5a–e). We used these data to generate DKL hue-direction maps (Fig. 6b, c and Supplementary Fig. 4d) using methods similar to those typically used to calculate orientation maps^43,44^; the hue-phase-regions from 12 hue-phase maps were selected, vectorized, and summed pixel-by-pixel to generate the hue-direction map. We then scaled the hue-direction map with the vectors’ magnitude to generate hue polar maps (Fig. 5g and Supplementary Fig. 5). The resulting hue-direction maps have a surprising and striking resemblance to orientation maps^43,45–48^ and motion direction maps^44,49,50^, including pinwheel-like structures (Fig. 6a–c, arrows highlight selected pinwheel centers) surrounded by linear iso-hue-direction regions. (See also orientation and hue polar maps in Supplementary Fig. 5). In some striking cases, the pinwheel centers correspond precisely to regions of convergence of L + M−/M + L− COFD pairs with S+/S− pairs whose axes are arranged orthogonally (Fig. 6a–c, dark arrows; note that Fig. 6b is generated by overlaying contours in Fig. 6a on top of Fig. 6c), but other pinwheel centers are found where COFD pair axes interact in a more parallel arrangement (Fig. 6a–c, white arrows), and some are even found outside the COFDs. DKL hue pinwheels were most apparent when generated using stimuli with cone increments maximized (Fig. 6a–c), but were also observed when cone increments were matched (Supplementary Fig. 4d).

### COFDs and hue preferences of individual neurons

We next assessed whether individual neurons within the COFDs prefer colors that would be predicted from the mixing of the cone-opponent signals within the COFDs. Figure 5f, g illustrates, for one imaging region (out of 5 sampled), the 2PCI-based functional microarchitecture for hue preferences in the DKL isoluminant plane (matched cone increments), and the overlaps with COFD maps are shown in Fig. 5h, i. (Maps from all five imaging regions, 10 planes, are shown in Supplementary Fig. 5). All visually responsive neurons are indicated, with significantly hue-selective neurons identified by their preferred hue and nonselective neurons in gray. It is readily apparent that neurons with similar hue preferences are grouped together and that hue preferences shift gradually across the cortical surface (Fig. 5g and Supplementary Fig. 5). The grouping of neurons preferring similar hues is demonstrated quantitatively by plotting the correlation coefficients of hue preferences against the distance between neurons (Fig. 5j). Correlations are far higher than a shuffled distribution at short distances (<100 μm) and fall off to near the shuffled distribution at larger distances. When overlaid on the M-cone COFDs, it is apparent that neurons in the M + (L−) domains prefer greenish hues while those in the M−(L + ) domains prefer pinkish hues (Fig. 5h). The predominantly red and greenish preferring neurons are both found in both S+ and S− domains (Fig. 5i).

To quantitatively evaluate the relationships between the preferred DKL hues of individual neurons and their locations within ISI COFD maps, we first selected neurons located within COFDs and that were significantly tuned to DKL hues (*n* = 2589 neurons from 5 imaging regions). We then generated a mask for each neuron to identify its corresponding ISI pixels, and the normalized pixel values from L, M, and S-COFD maps (see above) for each neuron were averaged to yield the magnitudes and signs modulated by L-, M-, and S-cone-isolating stimuli. Similar to Fig. 4j–l, three possible comparisons of cone types (M to L, S to L, and S to M) were plotted neuron-by-neuron (Fig. 5k–m), and each neuron was colored according to its preferred DKL hue measured by 2PCI. For the L-cone versus M-cone plot (Fig. 5k), pixels from the great majority of neurons (87.1%) are in the L/M opponent quadrants; neurons with pixels in the L + M− quadrant are significantly more likely to prefer DKL hues near 0° (0 ± 60°, 1167 of 1650 neurons, 70.7%) than near 180° (180 ± 60°, 462 of 1650 neurons, 28.0%; *P* < 0.0001, Fisher’s exact test, two-sided); and neurons with pixels in the M + L− quadrant are significantly more likely to prefer hues around 180° (180 ± 60°, 344 of 605 neurons, 56.9%) than around 0° (0 ± 60°, 250 of 605 neurons, 41.3%; *P* = 0.0018, Fisher’s exact test, two-sided). For the S-cone versus L and M-cone comparisons (Fig. 5l, m), neurons are more evenly distributed amongst the 4 quadrants, as expected from the overlap of both S+ and S− domains with all combinations of L- and M-cone domains.

The perceived color is influenced not only by the relative activation of different cone types, but also by luminance, or achromatic contrast. For example, the DKL color space (like other color spaces) includes modulation not only in the isoluminant plane, as we have explored here, but also along the achromatic axis^34^. As described above, we have observed achromatic ON and OFF ISI phase maps similar to the COFDs (Figs. 2i, j and 3e, i), and their overlap with COFDs suggests a possible role in the generation of hue preferences (Supplementary Fig. 3). But we have not explored the responses of individual neurons to the much larger 3D hue stimulus space. Future studies should explore how the achromatic ON and OFF domains might interact with the COFDs to generate predictable tuning across the full 3D color space.

### COFDs and DKL hue-direction maps

Consistent with 2PCI results indicating the mixing of cone-opponent signals to generate intermediate hue-selectivities of individual neurons within COFDs (above), we also observed gradual shifts in ISI hue-phase maps (Fig. 6d, e and Supplementary Fig. 4a–c) generated with stimuli modulated at non-cardinal directions in the DKL isoluminant plane (Fig. 5a). Gradual shifts of preferred color relative to cone-opponent domains indicate that the preferred colors at each location are the result of mixing of the cone-opponent signals within the overlapping COFDs. This contrasts with the alternative possibility in which the cone-opponent mechanisms do not mix, and therefore, intermediate color preferences would not be generated at the intersecting locations. These maps were generated using DKL stimuli with cone increments maximized (non-matched, see above) to better reveal the locations with responsiveness to S-cones. The gradual shifts in ISI hue-phase maps are quantified in Fig. 6d, e. Similar to comparisons between normalized ISI phase pixel values and signs across maps from the cone-isolating stimuli (Fig. 4j–l), each row of Fig. 6d illustrates comparisons between pixel values from each of the 12 hue-phase maps with either the L- (top row), M- (middle row), or S-cone (bottom row) phase maps. It can be seen that as DKL hue directions shift, the phase relationships to the COFD phases shift gradually (Fig. 6d, e and Supplementary Fig. 4b, c). When hue directions are near the L/M axis (0 and 180°), pixels are in opposing quadrants in relationship to the L and M phase maps (Fig. 6d and Supplementary Fig. 4b), and accordingly, the angles joining the two clusters of pixels in each plot are near 45 and 135° (Fig. 6e and Supplementary Fig. 4c). When these hue-phase maps (0 and 180° hues) are related to the S-cone phase maps, the pixels occupy all 4 quadrants (Fig. 6d and Supplementary Fig. 4b), and joining angles are near 90° (Fig. 6d and Supplementary Fig. 4c). As hue directions gradually increase in S-cone modulation, comparisons to the L and M phase maps show pixels gradually occupying all 4 quadrants (Fig. 6d and Supplementary Fig. 4b) and joining angles gradually shifting (Fig. 6e and Supplementary Fig. 4c), until at maximal S-cone modulation (90 and 270°) all 4 quadrants are occupied and joining angles are near 90°. In contrast, comparisons of hue-phase maps to the S-cone maps show pixels more evenly distributed across all 4 quadrants, with the exception of comparisons to the S+ and S− hues (90 and 270°) where opposing quadrants are occupied (Fig. 6d and Supplementary Fig. 4b) and joining angles are near 45 and 135° (Fig. 6e and Supplementary Fig. 4c). These observations indicate that not only does the overlap between COFDs follow the rules predicted to instantiate color appearance mechanisms (Figs. 1 and 4j–l), but that the interactions between L, M, and S-COFDs generate preferential responses to intermediate hues within the DKL color space, as predicted from those interactions (Figs. 5j–l, 6d, e, and Supplementary Fig. 4b, c).

### Cone inputs and hue tuning

For 2PCI-assayed neurons with significant STAs to the L-, M-, or S-cone-isolating stimulus set, their preferred hues in color space were also highly consistent with the magnitude and sign of cone inputs estimated from the STAs. This was the case both for hues presented in the DKL isoluminant plane (Fig. 5n) and in CIE color space^25^ (Supplementary Fig. 6a). This relationship is most apparent from the responses to DKL hues, due to the clear correspondence between cone-opponency and DKL hue directions. Normalized cone weight plots for both DKL and CIE hues (Fig. 5n and Supplementary Fig. 6a) show that the great majority of neurons are in the L + M− and M + L− cone-opponent quadrants, as expected from the mixing described above. Neurons in the achromatic quadrants have only weak STAs to one or another of the L or M-cone-isolating stimuli, as expected from the noise inherent to the calculation of input magnitude from STAs and the methods we used to assign values. (Neurons with a significant STA to only one stimulus set were nevertheless assigned values for the nonsignificant stimuli rather than pinning values to zero.) As expected, for the neurons tested with DKL hues, the great majority of hue-selective neurons in the L + M− quadrant preferred DKL directions around 0° (315 of 390 = 80.8% of neurons at 0 ± 60°), while those in the M + L− quadrant preferred DKL directions around 180° (119 of 213 = 55.9% of neurons at 180 ± 60°). For neurons tested with CIE hues, hue-selective neurons in the M + L− quadrant nearly all preferred blue, while neurons in the L + M− quadrant mostly preferred red, with some preferring blue (Supplementary Fig. 6a). This arrangement is expected from the stronger L-cone contrast for red and stronger M-cone contrast for blue (Supplementary Fig. 8c), combined with the fact that the OFF-phase of the blue stimulus can sometimes generate a stronger L response than M. It is important to note that hue-selective neurons generate both ON and OFF responses to drifting hue gratings and due to the slow dynamics of calcium signals observed with 2PCI, it is not possible to definitively identify whether responses are being generated to the ON versus the OFF phases of the drifting gratings. While ON responses are generally stronger than OFF responses, this may not always be the case. Therefore, the hue preferences observed from 2PCI imaging of responses to drifting gratings are not expected to always match the predictions from responses to flashed, cone-isolating stimuli.

There is also a clear separation of a set of neurons near the middle of the diamond plots with relatively strong S-cone STAs (Fig. 5n and Supplementary Fig. 6a). Note that the S-cone-isolating stimulus used to generate these STAs is much higher contrast (89.9–90.2%) than the L and M-cone stimuli (18–18.5%), contributing strongly to the separation of this group. This is apparent from the overall distribution of hue preferences to DKL stimuli (Fig. 5o). Here it can be seen that when stimuli are matched for cone-increment magnitude, responses are dominated by colors modulated at or near the L−M opponent axis (0 and 180°). Very few (138 of 6658 neurons, 2.07%) preferred stimuli modulated in the S+ or S− directions. Nevertheless, S-cone inputs could be seen to have a significant, although relatively weak, influence on preferred DKL hue. Amongst neurons with significant S-cone STAs, their preferred directions in the DKL isoluminant plane were consistent with expected shifts resulting from linear integration with L/M opponent signals. Neurons with significant S-cone ON STAs were more frequently tuned to the DKL hue at 90° than neurons lacking significant S-cone STAs (7 of 203 neurons, 3.45% versus 52 of 6167 neurons, 0.84%; *P* = 0.0058, Fisher’s exact test, two-sided), or than neurons with significant S-cone OFF STAs (0 of 288 neurons, *P* = 0.0045, Fisher’s exact test, two-sided). Similarly, neurons with significant S-cone OFF STAs were more likely to prefer the DKL hue at 270° than neurons without significant S-cone STAs (9 of 288 neurons, 3.13% versus 70 of 6167 neurons, 1.14%; *P* = 0.0184, Fisher’s exact test, two-sided), or than neurons with significant S-cone ON STAs (0 of 203 neurons, *P* = 0.0175, Fisher’s exact test, two-sided).

### Preferred hues within COFDs and their intersections

The relatively weaker influence of the S-cone relative to the L/M opponent signals during interactions between the COFDs can be seen not only in the hue tuning of the individual neurons (see above) but also in the tuning of ISI pixels within the different COFDs to the DKL stimuli. To further quantify the relationships between COFDs and DKL hue responses, the COFD masks were used to identify pixels in each COFD ON or OFF region (Fig. 6f and Supplementary Fig. 4e), as well as intersections between regions (Fig. 6g and Supplementary Fig. 4 f), and then the ISI hue responses of those pixels were used to generate hue tuning curves. For example, it can be seen in Fig. 6f that, as expected, the pixels in the L + and M− COFDs were most strongly modulated by hues at or near the 0° hue-direction and most weakly modulated by the 180° hue direction. In contrast, the M + and L− pixels were most strongly modulated at 180° and least strongly at 0°. Importantly these tuning curves are relatively symmetric around the 90–270° axis, as expected from near equal influences of S-ON and S-OFF signals, resulting from the symmetric mixing of S-ON and S-OFF COFDs with the L + M− and M + L− domains. Also as expected, the S + COFDs responded more strongly to 90° than to 270° and S− more strongly to 270° than to the 90° hue. But owing to the relatively weak S-cone influence, responses to the intermediate as well as 0 and 180° hues were at least as strong as those to 90 or 270°.

To more directly assess the predictions from the mixing of COFDs to generate color-opponent signals, we also generated DKL hue tuning curves for the pixels at the intersections corresponding to the regions where neurons would be expected to be shifted toward each of the red (L + ∩ S+), yellow (L + ∩ S−), blue (M + ∩ S+), and green (M + ∩ S−) color appearance mechanisms (Fig. 6g and Supplementary Fig. 4f). As expected from their L + contributions, the “red” (e.g., L + ∩ S+) and “yellow” pixels were strongly biased to preferences toward 0° and away from 180° hues. Similarly, the pixels corresponding to “colors” with M + contributions (“blue” and “green”) had DKL hue preferences that were strongly biased toward 180° and away from 0° Importantly, it can be seen that the differential mixing of S+ and S− for each of the combinations generates differential shifts toward or away from the S + (90°) and S− (270°) DKL hues. Specifically, the “red” pixels have hue preferences that are shifted toward 90° (away from 270°) relative to the “yellow” pixels, and the “blue” are similarly shifted relative to the “green” pixels. Again, despite the clear differences between the intersections with S+ versus S− COFD contributions, the shifts along the 90–270 axis are much smaller than the shifts along the 0–180° axis, once again reflecting the weak overall contributions of the S-cone inputs. This imbalance can also be visualized by comparing it to an idealized schematic that assumes balanced interactions across the COFD intersections (Fig. 6h) to combine cone-opponent signals. Figure 6h also serves to illustrate the hypothesized production of hue pinwheels at some locations where the physical positioning of COFDs generates interactions across orthogonal S+/S− and L + M−/M + L− axes (Figs. 4h, i and 6a–c). In such cases, the conceptualized schematic and the actual physical mapping of the brain have similar configurations.

### COFDs and CO histology

Previous studies have suggested that cytochrome-oxidase blobs in layer 2/3 are specialized for the processing of color information^27^ (but see refs. 13, 51), receive direct S+ input from the LGN^10,52^, and could have neurons with a different distribution of preferred colors than interblobs^25^. We were therefore interested in whether there might be a relationship between COFDs and CO staining, either for all COFDs together (data from all five animals) or for ON or OFF domains of particular cones (animal A7 excluded). Alignment of COFD maps to postmortem CO histology shows that they have similar periodicity and are often overlapping, but there are also clear regions that do not overlap (Fig. 7e, f, h and Supplementary Fig. 7). To quantify these relationships, the distributions of CO intensities were compared between pixels located within versus outside all COFDs. Complete results from all comparisons for all imaging regions are shown in Supplementary Fig. 7a. In general, regardless of the cone type (L, M, S) or phase (ON or OFF), pixels in the COFDs have higher CO intensities, and pixels in non-COFD regions have lower CO intensities compared to the overall distribution of CO intensities across V1 (Fig. 7g). The biases of these COFD regions toward high CO intensities were all statistically significant for every animal (Supplementary Fig. 7a, 4 animals for comparisons between phase-identified COFDs with non-COFDs, five animals for comparisons between phase-unassigned COFDs with non-COFDs). These statistical comparisons within animals are based on the treatment of each COFD region as an independent sample (Wilcoxon signed rank test, two-sided), and *P*-values less than 0.05 are considered significant. Across animals, the bias toward high CO intensities is statistically significant (*n* = 5 animals, *P* = 0.03125, right-tailed Sign test) only for the comparisons based on phase-unassigned COFDs (five animals), as maximal statistical significance for four animals is *P* = 0.0625. The achromatic ON and OFF domains were also significantly biased toward blob regions for all three animals in which achromatic phase maps were generated.

Because a previous study has shown that S + (L + M)− LGN afferents preferentially terminate in the CO blobs^10^, we were particularly interested in whether S + domains might be more strongly biased toward high CO intensity regions than other COFDs. Comparisons between cone types and phases within animals revealed consistent significant differences only for the S + domains, which were significantly more biased toward higher CO intensities than S− for all four animals (Wilcoxon rank sum test, two-sided, *P-*values less than 0.05 are considered significant, and the same test method and criteria were applied on the following comparisons). S + was also significantly higher than both L + and M− in three of four animals. Other comparisons were significant for only one or two out of the four animals. It is noteworthy that the densest S + LGN terminals and CO staining are found deep within layer 2/3 (layer 3B)^10,52^, so there might be a closer correspondence between S + responses and CO deeper in cortex.

We also quantified the relationships between CO staining and the preferred directions in DKL color space of neurons imaged with 2PCI (Fig. 7i). The highest CO staining intensities were observed at the locations of neurons preferring color directions around 180° (greenish) while the lowest CO intensities were observed at the locations of neurons preferring color directions around 0° (“pink”). These observations are consistent with the relationships previously reported between CO staining and color preferences of neurons tested using CIE colors^25^; neurons preferring green were observed to be at locations of higher CO staining intensity than those preferring red.

Previous studies have used ISI and colored visual stimuli to reveal patchy activation patterns (“color domains”) within V1^22,23^. We have generated ISI maps using the same methods applied in those studies (regions preferentially responsive to red/green or blue/yellow isoluminant stimuli) and directly compared those maps to COFDs imaged in the same animals. We find that the color domains and COFDs are closely related (Supplementary Fig. 7). This is expected, because both red/green and blue-yellow isoluminant modulation should differentially activate selected regions within the COFDs. The partial but not exclusive correspondence between COFDs and CO histology is also consistent with previous observations of the relationships between color domains and CO staining^21^.

## Discussion

In summary, we have demonstrated that: (1) L-, M-, S-COFDs, and achromatic ON/OFF domains are a prominent feature of the functional organization of primate V1 that can be readily revealed with ISI imaging; (2) 2PCI-assayed neurons within ON/OFF domains of L, M, S and achromatic have the same ON/OFF sign of that domain type; (3) both qualitative and quantitative assessment of the spatial intersections of COFDs shows that they follow the mixing rules predicted from the Three-stage model; (4) DKL hue-phase maps show that the hue preferences at intersections between COFDs correspond to hues predicted from functional mixing and generate hue pinwheel or linear structures; (5) 2PCI of DKL hue preferences at the intersections of COFDs shows that functional mixing occurs at the level of individual neurons; and (6) COFDs are biased toward regions of high CO intensity. We conclude that the neural substrates that underlie the mixing required for a transition from cone-opponent (Stage 2) mechanisms to color-opponent (Stage 3) mechanisms are highly organized and implemented by specific connections between neurons in the geniculate input layers (4 C, 4 A, 3B) and the neurons that we have assayed in more superficial V1.

The most prominent feature of Stage 3 in the Three-stage model is the specific mixing between cone-opponent mechanisms that are predicted to give rise to each of the four color-opponent mechanisms (Fig. 1a). This mixing is expected to generate a greater range of hue preferences than is present in the cone-opponent geniculate input population, largely owing to the interactions between the L/M and S/(L + M) opponent mechanisms that do not occur at earlier stages. That this mixing begins in V1 is expected from previous studies evaluating color preferences of V1 neurons^13^. The “unique colors” (red, green, blue, and yellow) are not necessarily expected to be preferred by larger numbers of neurons than other hues, but instead reflect perceptual transitions within the color-opponent mechanisms, where, for example, “red” appears neither “bluish” or “yellowish”^3^. (Due to the opponent nature of color appearance, red cannot appear “greenish”^31^.) Consistent with these expectations, our observations and analyses indicate that the mixing of L/M and S/(L + M) opponent signals within COFDs gives rise to shifts in preferred hues that are in the directions expected to generate color-opponent mechanisms. But the contribution of the S/(L + M) mechanism (S-cone contributions) is likely much less than expected to fully account for color appearance. Careful calculations based on psychophysical measures assign just a 2.55-fold stronger influence to the L/M mechanism^3^, while the distribution of color preferences we have measured from neurons in upper layer 2/3 of V1 appear to reflect a much stronger relative influence of the L/M mechanism (Fig. 5o). It is likely that these relative contributions are reweighted in higher visual areas, as suggested by a recent 2PCI study comparing responses to CIE hues in cortical areas V1, V2, and V4^29^. Importantly, our observations using DKL hues show that the biases of V1 neurons toward end-spectral CIE colors observed previously^25,29,53^ do not result solely from the stronger L and M-cone activation generated by the red and blue CIE hues^25,54^; S-cone contributions are weak even in response to DKL stimuli with matched L, M, and S-cone-increment magnitudes. Neurons deeper in layers 2/3 and 4 A (inaccessible to 2PCI) that receive direct S/(L + M) signals^10^ and can project directly to V2 might also have more dominant S-cone influences that could contribute to stronger S-cone influences in higher visual areas.

We were surprised to find that ISI mapping of the locations preferring DKL hues modulated in different directions (hue-direction maps) revealed “pinwheel” structures (Fig. 6) reminiscent of previously described orientation^43,45–48^ and direction maps^44,49,50^. Hue-direction pinwheels were surrounded by regions of iso-hue direction, again reminiscent of iso-orientation and iso-direction domains. Hue-direction pinwheels were often found at the intersections of L/M opponent and S-cone COFDs and, in the most striking cases, aligned with the intersections of orthogonal L + M−/M + L− and S+/S− COFD pairs (e.g., Figure 6a–c and schematized in Fig. 6h). Additional pinwheels were also located outside of the COFDs. We speculate that hue direction pinwheels emerge through developmental mechanisms that favor the grouping of neurons with similar functional preferences interacting with the requirement for retinotopic mapping (e.g., through wire length optimization^55^) and may share common principles with those underlying emergence of orientation and direction maps.

Electrode penetrations extending across cortical layers have also found locations containing neurons with Blue-Yellow versus Red-Green opponency, leading to the suggestion that there are separate blobs for each type of opponency^28^. The COFDs that we observe, as well as their relationships to CO staining, argue against this interpretation and provide an explanation for why different electrode penetrations might sometimes be biased to sample neurons with different color opponency. There may, in fact, be color columns that extend across cortical layers, but they likely correspond to the more closely spaced COFDs with different cone inputs rather than to separate blobs (see further below). Future experiments using ISI imaging of COFDs to guide subsequent electrode penetrations (e.g., Supplementary Fig. 9) should more clearly reveal the extent to which color selectivities and/or cone input signs and sensitivity extend across or vary between layers.

We have observed a functional organization (COFDs) in the most superficial layers of V1 that involves systematic mixing of L/M opponent with S+ and S− signals. The fact that L/M opponent LGN inputs terminate in a separate layer (layer 4Cβ) from S + (layer 3B) and S− inputs (layer 4 A) indicates that mixing of these cone-opponent systems can only occur through neural substrates that span across these layers and also suggest that there are likely to be some laminar differences in cone responses and color tuning. The most prominent substrate for potential mixing is the axons of layer 4Cβ spiny stellate neurons that extend to and densely arborize in layers 4 A and 3B, likely mixing L/M opponent with S-cone signals^36,56^. Yet another synaptic step is required to transmit information in layer 4 A/3B to the neurons that we imaged in layer 2/3A^35^. An additional possibility that should not be overlooked is a specialized population of layer 6 pyramidal neurons (Type IβA) that have both dendritic and axonal processes that arborize selectively in both layers 4Cβ and 4 A/3B^56–58^. These neurons could directly integrate L/M opponent with S+ or S− LGN inputs and provide their mixed signals to neurons in layers 4Cβ and 4 A/3B. Such a mechanism could account for Blue-Yellow opponent neurons that have been reported in layer 4C^28^. While our observations clearly point to these circuits as the neural substrates that mediate the mixing of cone-opponent mechanisms to generate the rudiments of color opponency, further experiments will be required to more precisely identify the micro-circuitry involved.

The V1 functional organization and micro-organization related to cone-opponency and color preferences that we have reported here build on a long history of prior studies using varied technologies and sometimes leading to apparently contradictory results. However, in light of the higher resolution and denser sampling afforded by 2PCI technology, sources of the apparent discrepancies are now much clearer. Before the development of ISI, studies used CO blobs as an anatomical landmark for linking the functional properties of sparsely sampled neurons to possible functional architecture. Notably, Livingstone and Hubel first reported a prevalence of neurons in and around CO blobs that were color-selective but not orientation selective, suggesting that color tuning would likely have some sort of functional organization. (But see ref. 53.) This was later reinforced by ISI studies demonstrating patchy cortical activation with isoluminant, full-field colored stimuli^22,23^. While the overall organization of color patches was similar to CO blobs with substantial overlap, there was clearly not a one-to-one correspondence. Those observations clarified that when single-unit recordings are related to blobs, blobs are an indirect and imperfect proxy for color functional architecture. Other studies combining single-unit recordings with postmortem CO histology provided the first evidence for a columnar organization for color preference and suggested that separate blobs might process “red-green” (L/M) versus “blue-yellow” (S/L + M) opponency^22,28^. But the first ISI maps of V1 locations preferring different colors suggested color transitions on a finer spatial scale than would be expected from separate L/M and S/(L + M) blobs^24^. This was confirmed when 2PCI was used to definitively identify functional microarchitecture for color preferences at the level of single neurons^25,26^. Neurons preferring similar colors are grouped together within a functional color microarchitecture, and neurons preferring different colors are spaced more closely than would be compatible with their separation by blobs.

Both Garg et al.^25^ and Chatterjee et al.^26^ also present results indicating that color mapping is dependent on mapping for spatial features, as first suggested by Livingstone and Hubel^27^. Chatterjee et al.^26^ show that color-selective neurons responding to full-field stimuli are strongly biased toward blobs, while those in the interblobs are relatively suppressed by full-field stimuli and instead respond well to drifting gratings that contain oriented edges. Garg et al.^25^ report that non-orientation selective, color-preferring neurons are strongly biased toward blobs, but orientation-selective color-preferring neurons are not.

Using CIE colors, Garg et al.^25^ also noted that neurons preferring green were biased much more strongly toward high CO regions than those preferring red or blue. Consistent with those observations, we find higher CO staining at locations with neurons preferring DKL hue directions around 180° (“greenish”) than 0° (“pink”). Here we also report that all of the regions within COFDs (generated with full-field stimuli) are significantly biased toward high CO activity (“blobs”) and that S-ON regions are significantly more biased than S-OFF COFDs. Altogether, these results point to an organization in which the blobs are a reliable marker of locations receiving direct S-ON input from the LGN^10,52^ and are part of a somewhat larger, overlapping system of COFDs. The COFDs mediate an orderly mixing of the S-ON input to blobs with L + /M− and M + /L− input from layer 4 Cβ and S-OFF input from layer 4 A, that follows the stage 3 mixing rules to generate a broader range of color preferences arranged in hue pinwheels.

## Methods

### Animals and animal care

All procedures involving live animals were conducted in accordance with the guidelines of the NIH and were approved by the Institutional Animal Care and Use Committee (IACUC) at The Salk Institute for Biological Studies. We recorded from seven adult macaque monkeys (*M. fascicularis*) under anesthesia in this study. They are referred as A1–A7. Details and allocation of these animals can be found in Supplementary Table 1. In brief, two-photon calcium imaging (2PCI) data were collected from A1, A3, and A4. Electrophysiology data were collected from A2 and A5. Intrinsic signal optical imaging (ISI) and histological data were collected from A1, A2, A5, A6, and A7.

### Surgery

Surgical procedures for AAV injections (recovery surgery), physiological recordings (nonrecovery), and euthanasia in this study are described below.

For animals used for 2PCI (A1, A3, and A4), two sequential surgeries were performed. First, a recovery surgery was conducted for the injection of AAVs to express the genetically encoded calcium sensor, GCaMP6f (see below). Then after 10–12 days of recovery to allow for GCaMP expression, a second nonrecovery surgery was performed, and an imaging chamber was implanted for ISI and 2PCI. For other animals used in this study, only the nonrecovery surgery was performed.

Before all surgeries, animals were anesthetized with ketamine (10 mg/kg) and pretreated with atropine sulfate (0.02 mg/kg) intramuscularly. During surgeries, animals were under general anesthesia (1–2.5% isoflurane in O_2_) and sterile conditions. Animals were intubated (tracheostomy was performed during nonrecovery surgery) and mechanically ventilated. EKG, SpO_2_, end-tidal CO_2_, and body temperature were all monitored throughout the procedure and during all recordings. Lactated Ringer’s with 5% dextrose, antibiotics (Cefazolin, 25 mg/kg, i.v.), and dexamethasone (1–2 mg/kg, i.v.) were supplied.

During the first surgery, following a craniotomy over V1, a 1:1 mixture of AAV5-TRE3-GCaMP6f and AAV5-thy1s-tTa^59^ (Salk Viral Core GT3, titer: 8.04E + 12 GC/ml and 2.13E + 12 GC/ml) was pressure injected via fine glass micropipettes at 1–3 depths in each of 6–12 locations in V1 (300–600 nl per injection, 300–700 µm below cortical surface). Injections were spaced about 1 mm apart to yield nearly continuous labeling. Following the injections, a thin piece of silicone artificial dura was placed between the cortical surface, and the natural dura, and these were glued in place with Vetbond (3 M, Maplewood, MN). The bone was replaced and cemented in place. Animals were recovered. Buprenorphine was administered for 3 days (slow release, 0.12 mg/kg, s.c.). Antibiotics (Cefazolin, 25 mg/kg, i.m., 1 week) and dexamethasone (1–2 mg/kg, i.m., 3 days) were administered daily to prevent infection and reduce brain swelling.

In the nonrecovery surgery, a head-post was implanted to stabilize the animal. A large imaging chamber (20 mm inner diameter) was cemented onto the skull over V1 and V2. A coverslip insert was screwed into the chamber to reduce brain movement. Wires were implanted through the small hole in the skull and connected to an amplifier to monitor EEG. Anesthesia was gradually transitioned from isoflurane to sufentanil citrate (2.0–8.0 µg/kg/h, i.v.), and vecuronium bromide (0.05–0.2 mg/kg/hr i.v.) was administered to paralyze the animal before recording, and EEG was subsequently monitored to continue along with other vital signs to assess anesthetic depth. Anesthesia dosage was adjusted at a level that insured slow-wave EEG activity. Eyes were dilated with 1% atropine sulfate and 0.5% proparacaine hydrochloride ophthalmic solution and fitted with contact lenses of appropriate curvatures to focus on a screen 57 cm from the eyes. First, we used ISI to obtain functional maps (e.g., ocular dominance map, orientation map, cone-opponent phase map, etc.) to target the regions of interest (e.g., cone-opponent functional domains) during 2PCI (on animals A1, A3 and A4, described below) or extracellular electrophysiology recording (on animals A2 and A5, described below). The animals were humanely euthanized by anesthetic overdose followed by perfusion at the end of the procedure (see “Histology and Image Alignment”).

### Intrinsic signal optical imaging (ISI)

Surface blood vessel images were acquired under 540 nm illumination. Images of reflectance change (hemodynamic signals corresponding to cortical activity) were acquired under 630 nm illumination. Imaging was performed using a GigE camera (Photonfocus, Switzerland) and custom Matlab software.

In this study, we conducted two ISI paradigms known as standard episodic stimulation combined with blockwise data acquisition and continuous-periodic stimulation combined with continuous data acquisition^42^. (see “Visual Stimulus Presentation”). For creating ocular dominance, orientation, and isoluminant color maps, we utilized the first ISI paradigm. Images were acquired during the pre-stimulus and stimulus periods (6 seconds, see below) at 10 Hz. For cone-opponent and DKL hue-phase maps, we utilized the second ISI paradigm, images were acquired at 10 Hz during stimulus presentation, which consists of a 2 sec pre-stimulus period and a 10–20 min stimulus period.

### Two-photon microscopy

Microscopy for two-photon calcium imaging (2PCI) was performed using a customized setup incorporating a Sutter movable objective microscope (Sutter Instruments, Novato, CA) with a resonant scanner (Cambridge Instruments, Bedford, MA) and data acquisition controlled by a customized version of Scanbox (Neurolabware, Los Angeles, CA). GCaMP6f was excited by a Ti:sapphire laser (Chameleon Ultra II, Coherent, Santa Clara, CA) at 920 nm. Of three animals used for 2PCI, two (A3 and A4) were single-plane continuously scanned at 15.49 Hz (unidirectional scanning); one (A1) was double-plane continuously scanned with an electrically tunable lens (Optotune, Switzerland) at 30.98 Hz (bidirectional scanning). An area of about 1168 × 568 µm (A3 and A4) or 1100 × 725 µm (A1) was imaged with a ×16, 0.8 NA objective lens (Nikon Corporation, Tokyo, Japan). The back aperture of the objective was not overfilled due to the technical limitations of the microscope.

### Extracellular electrophysiology

Extracellular electrophysiological data were collected using Neuropixels Phase 3 probes with 384 recording channels^60^. Briefly, signals were sampled at 30 kHz and filtered separately to an action potential band (0.3–10 kHz) and a local field potential band (LFP, 0.5–1000 Hz), then streamed to a computer under the control of SpikeGLX (http://billkarsh.github.io/SpikeGLX) software. The probe was held and lowered into the cortex using an oil hydraulic manipulator (Narishige International USA, Inc.), which was attached to the imaging chamber in order to ensure perpendicular penetration to the brain surface through a small hole in the coverslip. The locations of the electrode penetration were within cone-opponent functional domains (COFDs) revealed by ISI, and the probe was slowly lowered 2.5–3.1 mm below the brain surface. The brain surface at the location of the penetration was covered with 2% agarose and then with silicone elastomer (Sylgard 184, Dow Corning, USA) to prevent drying. Data were recorded at least 30 min later after the electrode was stable and visual fields were subsequently mapped.

### Visual stimulus presentation

Visual stimuli were generated with the Psychophysics Toolbox Version 3 (http://psychtoolbox.org/) for MATLAB (Mathworks Inc., MA) on a CRT monitor (CPD-G520, Sony Corporation, Japan) with a refresh rate of 100 Hz. For all experiments, the monitor was positioned 57 cm in front of the animal’s eyes. Prior to each experiment, the luminance of each of the red, green, and blue phosphors was linearized. Then a white point and mean luminance were chosen (*x* = 0.30, *y* = 0.31, *Y* = 71.5 cd/m^2^ for A1; *x* = 0.32, *y* = 0.34, *Y* = 86.3 cd/m^2^ for A2) as the background for spectra measurement of each phosphor with a spectroradiometer (PR-701, Photo Research, Syracuse, NY). Then we utilized the spectra of each phosphor to calculate gun gains for cone-isolating stimuli (including Hartley flash gratings and temporal-modulating gratings, see below) and DKL hue stimuli (see below, including drifting gratings and temporally modulated gratings, see below), and we used the same white point and mean luminance as background, interstimulus interval and blank condition during cone-isolating stimuli, DKL hue stimuli and full contrast achromatic grating stimuli (including drifting gratings and temporal-modulated gratings, see below) presentation. Another white point (*x* = 0.33, *y* = 0.33, *Y* = 10 ± 0.1 cd/m^2^ for other animals) was chosen as the background and blank condition for physical equal luminance CIE (Commission internationale de l’éclairage) hue stimuli^25^ (also referred to as CIE hues below).

For ISI experiments, both episodic and continuous-periodic stimuli were utilized. Episodic full-field achromatic square-wave drifting gratings (8 directions, 1.5 cycles/degree, 6.67 cycles/sec, duty cycle: 0.2) were presented binocularly to locate orientation columns and monocularly to obtain an ocular dominance (OD) map. Four CIE hues (red and green, blue and yellow) were chosen to construct Red/Green and Blue/Yellow isoluminant square-wave drifting gratings (4 directions, 0.2 cycles/degree, 2 cycles/sec, duty cycle: 0.5) to obtain Red/Green and Blue/Yellow isoluminant color maps. In order to increase the signal-to-noise ratio, each stimulus condition was presented in a randomized order with 20–30 repeats. For each trial, the stimulus was displayed for 4 sec with a 2 sec pre-stimulus period and 8 sec interstimulus interval. Continuous-periodic full-field cone-isolating (Fig. 3a), achromatic, and DKL hue temporally modulated gratings (Fig. 5a) were only modulated at a low temporal frequency (0.1, 0.08, or 0.017 Hz) to obtain cone-opponent, achromatic ON/OFF and DKL hue-phase maps. The temporal profile of these cone-isolating and achromatic stimuli was sine-wave (Fig. 3a); the temporal profile of DKL hue stimulus was square-wave with 0.5 temporal duty cycle (Fig. 5a).

For 2PCI, only episodic stimuli (drifting and Hartley flashed sine-wave gratings) were used, and all stimuli were presented monocularly. First, the dominant eye was selected based on the ISI ocular dominance map of the imaging area; the non-dominant eye was occluded. All stimuli were given through the dominant eye. Next, the receptive field of the imaging area was mapped by showing a series of drifting gratings, and the stimulus size (1.3–1.5° in diameter) was chosen to be as small as possible while still covering the receptive fields of all neurons within the imaging region.

Drifting gratings (achromatic, DKL hue, and CIE hue) were presented for 3–4 sec with a 3 to 4 sec interstimulus interval. Achromatic drifting gratings were presented as sine-wave gratings at 8 directions (0 to 315° in increments of 45°), 5 or 6 spatial frequencies (0.2, 0.4, 0.8, 1.6, 3.2, 6.4 cycles/degree), and a single temporal frequency of 5 cycles/sec. Each condition was repeated five times in random order. DKL and CIE hue drifting gratings were presented as square-wave gratings with 0.5 spatial duty cycle, 8 directions (same as achromatic gratings), 4 spatial frequencies (0.2, 0.4, 0.8, 1.6 cycles/degree), and a single temporal frequency of 5 cycles/sec. Each condition was repeated 3 times in random order.

The Hartley stimulus (flashed sine-wave gratings) set^37^ consisted of 120 blank stimuli and 5640 unique gratings with different combinations of orientation, spatial frequency (range: 0.2–6.0 cycles/degree), and spatial phase (range: 0, 90, 180, 270°) (Fig. 2a). The stimulus was presented for 60 trials (60 seconds per trial), and between each trial there was a 2 sec interstimulus interval. Within each trial, stimulus conditions were randomly selected from the stimulus set with replacement and were flashed continuously at 4 stimuli per second. A TTL pulse was generated at each stimulus transition and time-stamped with the corresponding microscope scanning frame and line number. The time stamps were used to synchronize stimulus and imaging frames for reverse correlation analysis (see “Spike-Triggered Average and Cone Weight Calculation”). The Hartley stimulus set was generated with L, M, S-cone-isolating colors and 98–99% contrast achromatic color.

For extracellular electrophysiology recording, a uniform patch was temporally modulated along two axes in the DKL isoluminant plane at 0.33 Hz with 0.5 temporal duty cycle (see “DKL Hues” below). The patch was presented monocularly to measure the ON/OFF responses to verify the ON/OFF phases (see “Intrinsic Signal Optical Imaging Data Analysis”) of the cone-opponent and DKL hue-phase maps.

### Cone-isolating calculation

To calculate cone-isolating directions^38^, we first calculated a 3 × 3 transformation matrix (*M*) by taking the dot product of the monitor spectral power distribution (*r*(*λ*), *g*(*λ*), *b*(*λ*). *λ* was sampled in the range [380, 780]nm every 2 nm) of the 10° cone fundamentals (*l*(*λ*), *m*(*λ*), *s*(*λ*)) published by Stockman and Sharpe^61^.

1 $$M=\left[\begin{array}{c}l\left(\lambda \right)\\ m\left(\lambda \right)\\ s\left(\lambda \right)\end{array}\right]\bullet \left[r\left(\lambda \right)g\left(\lambda \right)b\left(\lambda \right)\right]$$

Next, we utilized the following equation to calculate the R, G, B gun gains for each L-, M-, S-cone-isolating direction. Then, the gun gains for each direction were normalized to [−1 to 1].

2 $$\left[\begin{array}{c}R\\ G\\ B\end{array}\right]={M}^{-1}\bullet \left[\begin{array}{c}L\\ M\\ S\end{array}\right]$$

In all experiments, we matched the cone contrast of the L and M-cone-isolating stimuli and ran the S-cone-isolating stimuli at the maximum achievable contrast. The monitor was recalibrated prior to each experiment. Consequently, the exact contrasts of the cone-isolating stimuli changed slightly between experiments (L: 18.0–18.5%; M: 18.0–18.5%; S: 89.9–90.2%).

### DKL Hue

The background (center of DKL isoluminant plane) was the same background we used for measuring phosphor spectra (see “Visual Stimulus Presentation”). The same cone fundamentals used for cone-isolating stimuli were used for DKL hue calculation. Twelve hues were selected from the DKL isoluminant plane^62^ with equal increments from 0 to 330° (Fig. 5a and Supplementary Fig. 8b). For A1, the total cone-increment (pooled differential L-, M- and S-cone excitation between DKL hue and background) of each DKL hue was matched (Figs. 5f–i, 7h, Supplementary Figs. 4a, 4d, 5, 8b, and Supplementary Table 2); for A2, each DKL hue had the maximum achievable cone-contrasts by our display (Figs. 5b–e, 6b, c, Supplementary Figs. 4a, 8b, and Supplementary Table 2). Each cycle of spatial (for 2PCI) or temporal (for ISI) DKL hue square-wave gratings consists of a DKL hue and the background color with a 0.5 duty cycle. Except for this monopolar temporal modulation of DKL hues (modulation between DKL hues and the background) used for ISI, two bipolar temporal modulations of DKL hues (modulation between two DKL hues) were also applied for electrophysiology only. One of the bipolar modulations used two hues along L-M axis (0 and 180°, representing L + M− and M + L−, respectively) in DKL isoluminant plane. Another utilized two hues along S−(L + M) axis, which are 90 and 270° in DKL isolumiant plane, representing S + (L + M)− and (L + M) + S−, respectively.

### CIE Hues

The CIE hue stimuli were identical to those described in a previous publication^25^; more details of the stimuli can be found in the supplementary materials of that paper. Briefly, twelve hues were selected from the HSL plane (S = 100%, L = 50%) with an equal increment from 0 to 330°. Then the CIE-1931 *xyY* coordinates (Supplementary Fig. 8a and Supplementary Table 2) of each hue were measured with a spectroradiometer, and we adjusted the R, G, B value of each hue to match their luminance (*Y*) to 10 ± 0.1 cd/m^2^. Because of this adjustment and the gamut of the CRT monitor, the twelve hues were not in the same plane of the HSL color space, but they had the same luminance (*Y*). The CIE *xy* coordinates of each hue remained the same between different experiments. For A3 and A4, we presented the CIE hues. One cycle of the CIE hue spatial square-wave gratings consists of a CIE hue and the background color (*x* = 0.33, *y* = 0.33, *Y* = 10 ± 0.1 cd/m^2^, see “Visual Stimulus Presentation”) with 0.5 duty cycle.

### Histology and image alignment

Animals were euthanized by a lethal dose of pentobarbital sodium (Euthasol, i.v.), and transcardial perfusion was performed using 0.9% saline, followed by 4% paraformaldehyde (PFA), 10% sucrose with PFA, and 20% sucrose with PFA, and the brain was extracted. The recording region was then blocked, flattened, and sunk in 30% sucrose and sectioned (50 µm) tangentially using a freezing microtome. Tissue was stained for cytochrome-oxidase (CO), and sections were imaged. Both CO and 2PCI images were aligned manually with the ISI blood vessel map in Adobe Photoshop. Regions from ISI images corresponding to 2PCI images were interpolated in Adobe Photoshop using ‘Bicubic Automatic’ method.

### Intrinsic signal optical imaging data analysis

Data was analyzed by customized software written in MATLAB. For the episodic stimulation paradigm, the percent change (∆*R*/*R*) of each pixel for each stimulus condition was first calculated using the following formula:

3 $$\frac{\triangle R}{R}=\frac{R-{R}_{0}}{{R}_{0}}$$

*R* is the average response of a pixel during the stimulus period, and *R*_0_ is the average response of a pixel during the pre-stimulus period. The ∆*R*/*R* image of each condition was then filtered using a fourth-order Butterworth bandpass filter (high cutoff frequency: 7.8–11.2 cycle/mm, low cutoff frequency: 0.8–2.1 cycle/mm). Next, we computed a t-map^44^, and based on the *∆R*/*R* images of two conditions to reveal the OD columns and orientation columns. The orientation polar map (from t-maps, Supplementary Fig. 5), DKL hue direction (from DKL hue-phase maps, Fig. 6b, c and Supplementary Fig. 4d), and DKL hue polar map (hue-direction map scaled by vector magnitude pixel-by-pixel, Fig. 5g and Supplementary Fig. 5) were calculated using vector summation method^43,44^. For the continuous-periodic stimulation paradigm, phase map computation has been described^42^. Specifically, the *∆R*/*R* image of each camera frame during the stimulus period was first calculated to reduce noise, then the phase of each pixel was extracted from the ∆*R*/*R* image sequence to plot the phase map. Finally, the phase map was clipped to ±1.5 SD (standard deviation) of the image, and then filtered using the contrast-limited adaptive histogram equalization (CLAHE) method provided by MATLAB. Hemodynamic delay in the phase map was not compensated^42^; instead, we took advantage of the results from 2PCI or electrophysiology recording to identify the sign of ON/OFF phases in the ISI phase maps (Figs. 3, 4, 5h, i, 7a–c, and Supplementary Figs. 1, 2b, 3a–b, 9b).

### Image processing for COFD contours, histograms, and ISI DKL hue tuning curves

To further investigate the spatial relationship between different COFDs and between COFDs and DKL hue domains, COFD contours, bivariate histograms, and the DKL hue tuning curve of each COFD were plotted. To plot COFD contour maps, COFD phase maps were first filtered with a 2-dimensional Gaussian kernel (σ = [5, 5]), then contours were extracted from filtered images to generate COFD contour maps. Contours corresponding to the highest 15% and lowest 15% of values correspond are used to illustrate ON or OFF regions (Figs. 4d–h, 6a, b, 7e, f, and Supplementary Figs. 2a, 3b, c, 7a, b, 9b).

To plot bivariate histograms, COFD masks were generated by selecting contour level values that best corresponded to the domain sizes of each phase map. We manually removed some regions (e.g., blood vessel, noise) to make sure the masks are specific to regions of interest. A blood vessel mask and area mask were also added to exclude pixels within blood vessel regions or other areas outside V1 (e.g., V2). Next, COFD phase maps and DKL hue-phase maps were filtered with a two-dimensional Gaussian kernel (σ = [5, 5]) and normalized to [−1 to 1]. Then pixels on COFD maps were selected based on the combined L-, M-, S-cone COFD masks to plot bivariate histograms to show spatial relationships between different COFD maps (Fig. 4j–l and Supplementary Fig. 2a). Similar histograms were also plotted to show the spatial relationship between COFD maps and Achromatic ON/OFF map (Supplementary Fig. 3d–f). Pixels on COFD maps and DKL hue-phase maps were selected based on each L-, M-, S-cone COFD mask to plot bivariate histograms to show spatial relationship between COFDs and DKL hue domains (Fig. 6d and Supplementary Fig. 4b). All bivariate histograms have 50 × 50 bins; bin counts of each histogram were normalized to [0 to 1]. Specifically, for each COFD-DKL hue bivariate histogram, the center of the top 10% (brightest) pixels was calculated independently in the upper and lower quadrants. Then, the angle of the line connecting the centers in the upper and lower quadrants was calculated to show that L-, M-, and S-COFDs have different and systematic spatial relationships with DKL hue domains (Fig. 6e and Supplementary Fig. 4c). Curves were fitted using MATLAB fit function with smoothing spline model.

To plot the DKL hue tuning curve of each COFD, DKL hue-phase maps were filtered with a two-dimensional Gaussian kernel (σ = [5, 5]) and normalized to [0 to 1]. The mean pixel value of each DKL hue-phase map was calculated based on each L-, M-, and S-cone COFD mask (Fig. 6f and Supplementary Fig. 4e) or based on the intersection regions of COFD masks (Fig. 6g and Supplementary Fig. 4f). Curves were fitted by Generalized von Mises function (*GvM*) as described by the following equation^63,64^:

4 $${GvM}\left(\theta \right)=A{e}^{{\kappa }_{1}\left({\cos }\left(\theta \,-\,{{{{{{\rm{\mu }}}}}}}_{1}\right)\,-1\right)+{\kappa }_{2}\left({\cos }\left(2\left(\theta \,-\,{{{{{{\rm{\mu }}}}}}}_{2}\right)\right)\,-1\right)}$$

where *θ* is the DKL hue direction in the range of [0 2π); ${\mu }_{1}\in$[0 2π) and ${\mu }_{2}\in$[0 π) are preferred directions; ${\kappa }_{1}$, ${\kappa }_{2}$ > 0 are the measure of concentration about μ_1_ and μ_1_.

To plot the spatial relationship between CO intensity and COFD maps, blood vessel holes in CO images were first filled using ‘Content-Aware’ method in Adobe Photoshop. Then CO images were filtered with a 2-dimensional Gaussian kernel (σ = [5, 5]) and normalized to [0 to 1] with a higher pixel value means higher CO intensity. Pixels within COFD regions (combined L-, M-, S-cone COFD masks) and non-COFD regions were selected (pixels within blood vessels were excluded) to plot the histogram (Fig. 7g and Supplementary Fig. 7a). If there was bleach of CO intensity in CO image due to laser scanning or electrode penetration (e.g., Fig. 7d), pixels within that region were excluded for quantitative analysis. After aligning DKL hue preference map with CO image (Fig. 7h), pixels corresponding to neurons preferring the same hue were grouped, and the CO intensity distribution of that group was plotted as 4 levels (0–25%, 25–50%, 50–75%, and 75–100%) in one column shown in Fig. 7i. Pixels within each COFD and achromatic ON and OFF mask, and within whole V1 region were selected and grouped into 4 levels (0–25%, 25–50%, 50–75%, and 75–100%) based on CO intensity levels, see Supplementary Fig. 7a. For animal A7, only histograms of COFD regions versus non-COFD regions were plotted because we did not identify the ON/OFF-phase of the COFDs from that animal (Supplementary Fig. 7a).

### Two-photon calcium imaging data analysis

Regions of interest (neuron cell bodies) were manually segmented using Adobe Photoshop. Raw fluorescence signals were extracted, and the spike rate was estimated by Scanbox^65^ (https://scanbox.org/). For two-plane bidirectional scanning, raw fluorescence signals were interpolated before spike estimation. Thus, interpolated signals and spikes had 30.98 Hz sampling rate for both planes. Once signals and spikes were extracted, they were analyzed using customized software written in MATLAB and The Julia Language (https://julialang.org/). The change in fluorescence *(*∆*F/F*) was computed by the following equation, where *F* is the average fluorescence during stimulus presentation and ${F}_{0}$ is the average fluorescence during the pre-stimulus period:

5 $$\frac{\triangle F}{F}=\frac{F-{F}_{0}}{{F}_{0}}$$

Responses were averaged over 3–5 stimulus presentations, and the stimulus condition (e.g, direction, spatial frequency and hue) of the maximal response was identified. Neurons were considered visually responsive if they had a *P* < 0.05 to either two-way ANOVA across all stimulus conditions (including blank stimuli), or two-tailed Welch’s *t*-test between the strongest response and response to the blank stimulus.

To determine whether a neuron was significantly direction and/or orientation tuned, we chose all direction conditions at a neuron’s preferred spatial frequency. Then we applied the circular variance method^66,67^ to average the responses of eight direction to get one direction vector. Each condition was repeated five times in the experiment, so there were five direction vectors for each neuron. Similarly, five orientation vectors were obtained by averaging two directions of motion at each orientation. Then we projected the five vectors onto their final mean vector, thus the two-dimensional distribution of 5 vectors was transformed to a one-dimensional distribution. Note that each of those five vectors depended on how the conditions combined as one repeat, therefore the condition combination affected the distribution of five vectors. To reduce the distribution bias introduced by low sampling, we randomly shuffled direction or orientation conditions 100 times and calculated the vectors to produce 500 data points demonstrating the distribution of the direction or orientation response magnitude of a neuron. We did the same procedures for the blank condition and calculated the distribution of the blank response magnitude of the same neuron. Then we analyzed the two distributions using ROC analysis^68^ and calculated the probability of the area under a ROC curve (AUC, used the trapezoidal rule) as a parameter to indicate the significance of direction or orientation selectivity. If AUC > 0.7, we considered the neuron to be direction or orientation selective. The preferred direction or orientation is the angle of the final mean vector from the circular variance calculation (Supplementary Fig. 5).

To determine whether a neuron was significantly hue tuned, we first tested whether the neuron was direction and orientation tuned as described above, but chose all directions using the spatial frequency and hue to which the neuron was maximally responsive. If a neuron was direction or orientation tuned, all hue conditions were chosen under the neuron’s preferred spatial frequency and direction or orientation. Otherwise, hue conditions were chosen under the neuron’s preferred spatial frequency for circular variance and ROC analysis, similar to the determination of direction and orientation selectivity. The AUC was calculated based on the ROC curve of distributions of blank and hue conditions. If AUC > 0.8, we considered the neuron to be hue selective, and the preferred hue was the hue to which the neuron maximally responded (Fig. 5f and Supplementary Fig. 5).

To show the relationship between neurons’ DKL hue preference and COFD organization, neurons within COFD regions were plotted as dots labeled with their preferred hues with normalized L-, M-, and S-cone COFD magnitude as coordinates (Fig. 5k–m). First, 2PCI images were aligned with the ISI blood vessel map, and regions of ISI images corresponding to 2PCI images were interpolated (see “Histology and Image Alignment”). Then, interpolated ISI images were filtered with a 2-dimensional Gaussian kernel (σ = [20, 20]) and normalized to [−1 to 1]. Next, pixels on L-, M-, and S-cone COFD maps were selected based on the neuronal segmentation mask to calculate the normalized COFD magnitude for pixels corresponding to each neuron. Neurons within COFD regions from 5 imaging regions (10 planes) of A1 were selected to plot neuronal COFD magnitude distribution with their preferred DKL hues (Fig. 5k–m).

### Spike-triggered average and cone weight calculation

First, we used the time stamps to synchronize the stimulus sequence and estimated spike trains (Fig. 2). Then, the linear kernel of the receptive field was estimated by spike-triggered average (STA) using the following formula modified from previous publication^69^:

6 $${\hat{k}}_{{ls}}={({X}^{T}X)}^{-1}{X}^{T}Y={{cX}}^{T}Y=\alpha {k}_{u}$$

where ${\hat{k}}_{{ls}}$ is the estimated kernel using least-square, *Y* is a column vector of neural responses to each unique stimulus condition been presented and *X* is the stimulus matrix with each row is linearized unique stimulus. All 4 Hartley stimulus sets (L-, M-, and S-cone-isolating, and achromatic) share the same Hartley basis function, which is radially symmetric, therefore, ${({X}^{T}X)}^{-1}$ can be simplified as constant*c*, which is the same for all Hartley stimulus set. Note that this constant*c* is canceled when calculating magnitude change and does not affect the normalized cone weights calculation neither (see below). The estimated kernel ${\hat{k}}_{{ls}}$ is a 2D image, which is also a vector in multidimensional space with each pixel as a dimension. The vector ${\hat{k}}_{{ls}}$ can be represented as its magnitude (*α*) multiples with its unit vector ($\,{k}_{u}$). STA was calculated using 33 ms (bidirectional scanning) or 66 ms (unidirectional scanning) time bins of estimated spikes within a time window (−400 to 66 ms). In this STA movie, we defined a neuron’s best kernel estimation (${k}_{{u{{{{{\mathrm{best}}}}}}}}$) as the time when the magnitude reached its peak (${\alpha }_{{\max }}$), and this time was defined as the best time delay. Typically, this occurred 198–300 ms prior to a spike, which is very similar to the time window reported in mouse visual cortex^70^. To check the significance of${k}_{{u{{{{{\mathrm{best}}}}}}}}$, we defined the kernel estimated in the time window (0–66 ms) after each spike as ‘blank’ kernel (${k}_{{u{{{{{\mathrm{blank}}}}}}}}$) with its magnitude (${\alpha }_{{{{{{{\mathrm{blank}}}}}}}}$), then z-scored${k}_{{u{{{{{\mathrm{best}}}}}}}}$ was computed based on${k}_{{u{{{{{\mathrm{blank}}}}}}}}$. The ${k}_{{u{{{{{\mathrm{best}}}}}}}}$ was considered significant if it passed all three criteria: (1), its ${\alpha }_{{\max }}$ must within 198–300 ms time window prior to a spike; (2), its magnitude change (∆α/α, defined below) must bigger than 0.25 threshold; (3), the variance in z-scored ${k}_{{u{{{{{\mathrm{best}}}}}}}}$ was 3.5× greater than the variance of z-scored${k}_{{u{{{{{\mathrm{blank}}}}}}}}$, or the absolute mean of z-scored ${k}_{{u{{{{{\mathrm{best}}}}}}}}$ was 3.5× greater the absolute mean of z-scored ${k}_{{u{{{{{\mathrm{blank}}}}}}}}$.

7 $$\frac{\triangle \alpha }{\alpha }=\frac{{\alpha }_{{\max }}-{\alpha }_{{{{{{{\mathrm{blank}}}}}}}}}{{\alpha }_{{{{{{{\mathrm{blank}}}}}}}}}$$

A neuron had four best STAs calculated from L-, M-, S-cone-isolating and achromatic Hartley stimulus sets, respectively; if any of four STAs passed those three criteria, this neuron was considered as having a kernel that can be estimated by the STA of that stimulus set. A neuron could have one or more significant STAs from different Hartley stimulus sets.

If a neuron has a significant STA to a Hartley stimulus set, we obtained the sign (positive or negative) of the maximal absolute value in its best STA, and used the sign to plot the ON (positive) and OFF (negative) maps (Figs. 2, 3, and Supplementary Fig. 1).

To calculate the normalized cone weights in Fig. 5n and Supplementary Fig. 6a, only neurons with a significant STA to the L-, M-, or S-cone-isolating Hartley set were included. For each neuron in this group, we first compared the ${\alpha }_{{\max }}$ from three cone types, and we defined the dominant cone type as the one with maximal${\alpha }_{{\max }}$. Then, we used the best time delay of the dominant cone type to slice out three images (${k}_{u}$) from L-, M-, and S-cone STA movies. Next, we made a mask by thresholding the top 5% of absolute pixel values in the image of the dominant cone type, and used the same mask on all three images to select pixels to calculate their mean value. The mean values from L-, M-, and S-cone images are the raw cone weights (${{w}_{l}{,w}_{m},w}_{s}$, respectively). The normalized L-cone weight (${W}_{l}$) was calculated using the following equation, and similarly for the normalized M- and S-cone weight (${W}_{m}$ and${W}_{s}$).

8 $${W}_{l}=\frac{{w}_{l}}{\left|{w}_{l}\right |+\left|{w}_{m}\right |+{w}_{s}\vee }$$

In the L-M-cone weight plots provided (Fig. 5n and Supplementary Fig. 6a), the S-cone weight is implicit in the distance from the diagonal lines. We chose neurons with a significant STA to S-cone-isolating Hartley set, and plotted the histograms based on the ON/OFF signs to S-cone stimuli and their preferred hues (Fig. 5o–q and Supplementary Fig. 6b–d).

### Extracellular electrophysiology data analysis

Data were analyzed using customized software written in The Julia Language. The LFP gamma band power has been reported to be closely related to hemodynamic signals recorded by ISI^71^. Therefore, we utilized the LFP gamma band power to identify the ON/OFF phases of the COFD phase map. The LPF band (0.5–1000 Hz, see “Extracellular Electrophysiology”) was bandpass filtered (1–100 Hz), and 60 Hz line noise was removed. Then, LPF power spectrums were estimated by Multi-Taper methods from 0–300 ms following and −300 to 0 ms before stimulus onset, which corresponds to the response (${P}_{r}$) and baseline (${P}_{b}$) epochs. Stimulus responses were defined as relative changes in the power spectrum (${P}_{{rc}}$) within these epochs using the following equation.

9 $${P}_{{rc}}=\frac{{P}_{r}-{P}_{b}}{{P}_{b}}$$

The gamma band power (30–100 Hz) of the recording channels within the superficial layer (0–300 μm) was chosen to identify ON/OFF phases of the COFD within which electrode penetrations were made (Supplementary Fig. 9).

### Statistics and reproducibility

All cases, including the repetition of experiments, are summarized in Supplementary Table 1. In all statistical tests, *P* < 0.05 was considered significant. The visual responsiveness of neurons was tested using two-way ANOVA and two-tailed Welch’s *t*-test. The distributions of neuronal DKL hue preferences were compared using Fisher’s exact test.

To statically compare the CO intensity distribution within COFD regions versus non-COFD regions, COFD and non-COFD regions were selected using the masks described above. For within animal comparisons, each COFD region was treated as an independent sample, which was represented as the median of the CO intensity within that COFD region. For each animal, the CO intensity median values (*x*) within COFD regions were compared with the median (*y*) within non-COFD regions to test whether (*x*-*y*) has zero median against the alternative hypothesis that the median is not zero using Wilcoxon signed rank test (two-sided). For comparison across animals, we applied Sign test (right-tailed) to see whether the difference between the median of CO intensity within all COFDs and non-COFD is significant across animals. The same Wilcoxon signed rank test (two-sided) was also performed on achromatic ON/OFF regions versus non-COFD regions. For animals in which we identified the ON/OFF sign of ISI phase map, we compared the CO intensity distribution in ON regions versus OFF regions within and between each cone type in each animal. The L-, M-, and S-COFD masks were the same as described above. We performed Wilcoxon rank sum test (two-sided) to check whether two distributions have equal medians.

To test the significance of overlaps between different COFDs measured with ISI, normalized distributions of pixel densities from overlaps of COFDs for each combination of two cone types (e.g., Fig. 4j–l) were collapsed into two histograms (*n* = 20 bins) corresponding to overlaps in the same-sign and in the opposite-sign quadrants (cf. Figure 4j–l, Supplementary Fig. 2a and Fig. 3d–f). The distributions of these two histograms were then compared using a two-sample Kolmogorov–Smirnov test (two-sided).

### Reporting summary

Further information on research design is available in the Nature Research Reporting Summary linked to this article.

## Supplementary information

Supplementary Information

Reporting Summary

## Data Availability

All data necessary to support the paper’s conclusions are present in the main text, supplemental information, or available from the corresponding author upon reasonable request. The results presented here are based on more than 5TB of raw data and include data measuring activity during stimulus conditions that are not relevant to the present manuscript. The authors are presently conducting further analyses of that data which will be published at a later time. These data are not structured to allow data relevant to this manuscript to be separated from that which is reserved for future publication. Source data are provided with this paper.

## Source data

Source Data

## Code availability

Customized code used to perform the analyses can be found at https://github.com/Peichao/COFD_Nature-Commun and https://github.com/Experica.
