# Supplementary material for: Cone opponent functional domains in primary visual cortex combine signals for color appearance mechanisms
Source: Nat Commun. 2022 Oct 25;13:6344. doi: 10.1038/s41467-022-34020-2 (PMC9596481; doi:10.1038/s41467-022-34020-2)
Supplement: Supplementary file 1 — Supplementary Information [file 41467_2022_34020_MOESM1_ESM.pdf]

Title: Cone Opponent Functional Domains in Primary Visual Cortex Combine Signals for Color Appearance Mechanisms

Authors: Peichao Li[1] *[†], Anupam K. Garg[1,2,3]*[††], Li A. Zhang[1], Mohammad S. Rashid[1] and Edward M. Callaway[1,2]

Affiliations:

[1]The Salk Institute for Biological Studies, La Jolla, California, 92037

[2]Neurosciences Graduate Program, University of California, San Diego, La Jolla, California, 92093

[3]Medical Scientist Training Program, University of California, San Diego, La Jolla, California, 92093

†Present Address: Liangzhu Laboratory, MOE Frontier Science Center for Brain Science and Brain-machine Integration, State Key Laboratory of Brain-machine Intelligence, Zhejiang University, 1369 West Wenyi Road, Hangzhou 311121, China

††Present Address: Wilmer Eye Institute, Johns Hopkins University, 600 N Wolfe Street, Baltimore, Maryland, 21287

*These authors contributed equally.

Correspondence: callaway@salk.edu.

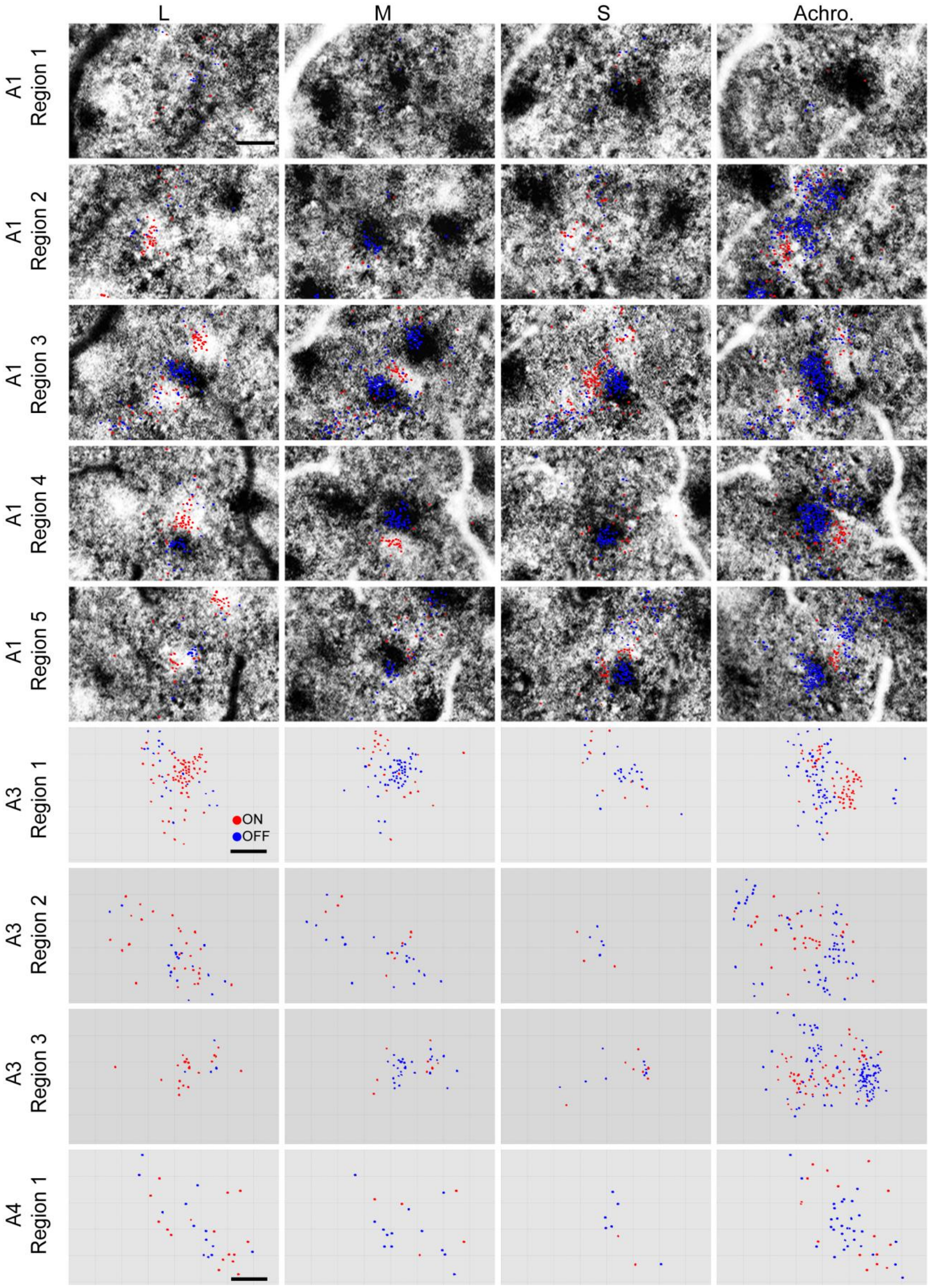

L
M
S
Achro.
A1 Region 1
A1 Region 2
A1 Region 3
A1 Region 4
A1 Region 5
A3 Region 1
A3 Region 2
A3 Region 3
A4 Region 1
ON
OFF

Supplementary Fig. 1 Additional cases of ON/OFF-dominant achromatic and cone-opponent receptive field clusters.

Each row corresponds to one imaging region with neurons (dots) from two planes merged. Each column corresponds to responses to one type of cone-isolating (L, M, and S) or achromatic (Achro.) stimulus. Animals and imaging regions that were the source of the data are indicated to the left of each row. The first 5 rows are imaging regions from A1, with 2PCI neuron properties overlain on ISI COFD phase maps. "A1 Region3" is the same region shown in Figs. 2i, 3f-i, 5f-i, and 7h. "A1 Region4" is the same region shown in Fig. 2j. Red dots represent ON-dominant neurons; blue dots represent OFF-dominant neurons. Scale bars: 200 µm; the three scale bars each apply to all additional panels from same animal. Repetition of these experiments is summarized in Supplementary Table 1.

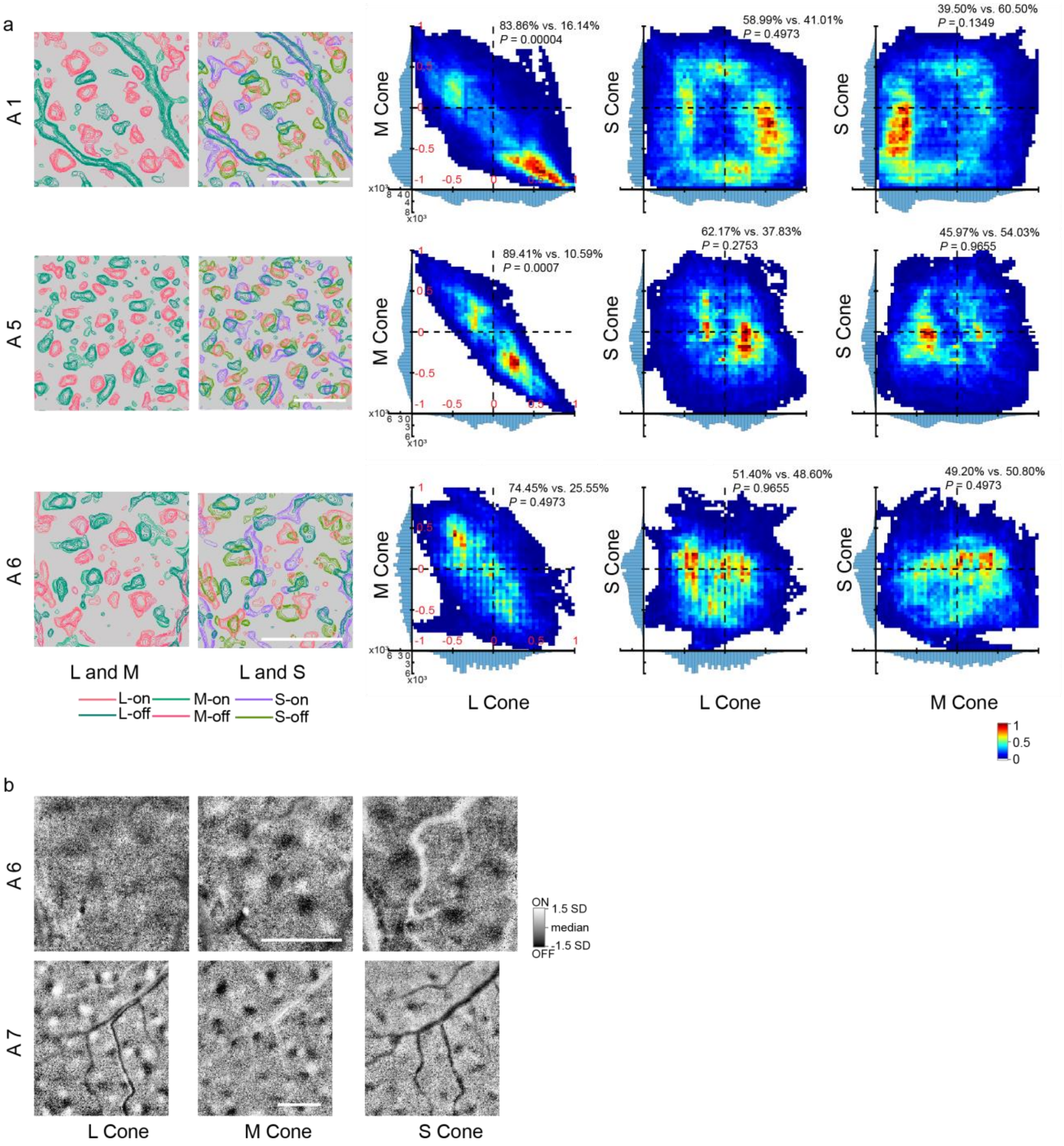

Supplementary Fig. 2 More cases of COFD maps and their spatial organization.

a, Each row consists of results from one animal. The COFD spatial organization and their relationship has been demonstrated in 4 ISI cases. One is shown in Fig. 4 (A2), others are shown here (A1, A5 and A6). First column is the overlay of L-cone and M-cone COFD contours. Second column is the overlay of L-cone and S-cone COFD contours. Scale bar for each case: 1 mm. Last three columns are the quantitatively analysis of the spatial relationship between L, M and S COFDs shown as bivariate histograms. The ratio and the *P* value (Kolmogorov-Smirnov test, two-sided) of pixel distributions between opposite-sign quadrants vs. same-sign quadrants are shown on the top of each histogram.

b, Two more cases of COFD maps. For A7, we did not identify the ON/OFF phases of pixels in three maps. Scale bar for each case: 1 mm.

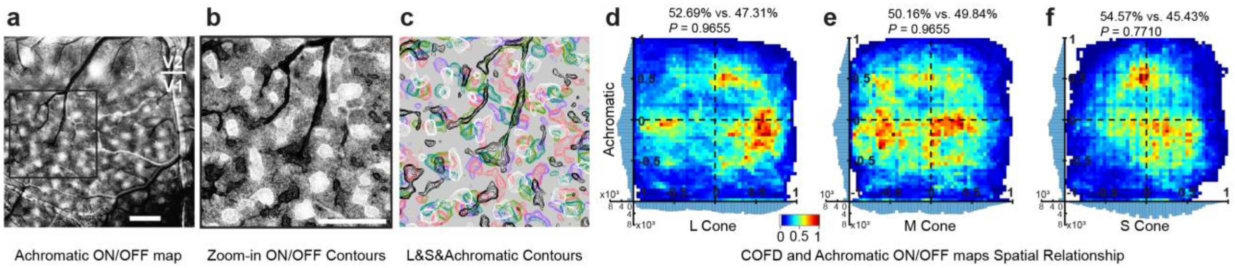

Supplementary Fig. 3 Achromatic ON/OFF phase map and its spatial relationships with COFDs.

a, Achromatic ON/OFF map. Note that this is the same cortical region as shown in Fig. 4a-c. Scale bar: 1mm.

b, Zoom-in view of the region selected in **a**, and overlaid with ON/OFF contours. Note that this is the same cortical region as shown in Fig. 4d-f. Scale bar: 1mm; applies to **b** and **c**.

c, Overlays of the L- and S-COFD contours with Achromatic ON/OFF contours.

d-f, Bivariate histograms of the spatial relationship between COFDs and Achromatic ON/OFF map. The 1D histograms on the *x* and *y* axes display the distribution of pixel values in each category. Similar to Supplementary Fig. 2a, the ratio and the *P* value (Kolmogorov-Smirnov test, two-sided) of pixel distributions between opposite-sign quadrants vs. same-sign quadrants are shown on the top of each histogram.

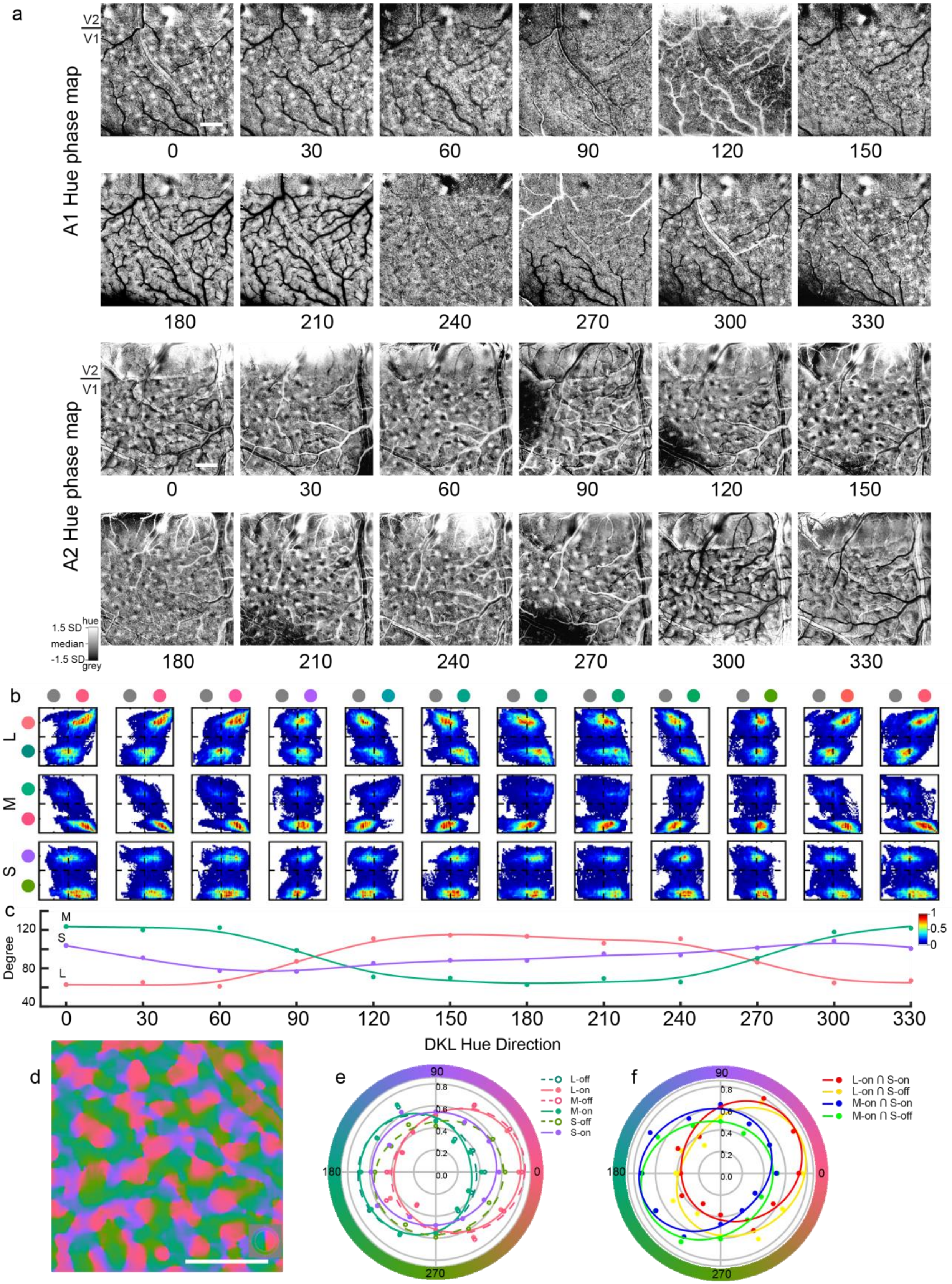
a
A1 Hue phase map
V2
V1
0
30
60
90
120
150
180
210
240
270
300
330
A2 Hue phase map
V2
V1
0
30
60
90
120
150
180
210
240
270
300
330
1.5 SD
median
-1.5 SD
hue
grey
b
L
M
S
c
M
S
L
Degree
120
80
40
1
0.5
0
0
30
60
90
120
150
180
210
240
270
300
330
DKL Hue Direction
d
e
90
180
0
270
L-off
L-on
M-off
M-on
S-off
S-on
f
90
180
0
270
L-on ∩ S-on
L-on ∩ S-off
M-on ∩ S-on
M-on ∩ S-off

Supplementary Fig. 4 All DKL hue phase maps and their spatial relationship with L-, M-, and S-COFDs.

a, All DKL hue phase maps from two animals (A1 and A2). The number under each image is the hue direction in DKL isoluminant plane. The DKL hues used for A1 were cone-increment-matched, in which S-cone modulation (90 and 270 degree) is weak, but for A2, cone contrast of each hue was maximized on the monitor; therefore, signals in A1 were weaker, especially along S-axis (90 and 270 degree). Scale bars in two cases are 1 mm.

b, The bivariate histograms show systematic relationship between COFDs and DKL hue preference domains in animal A1. Same to Fig. 6d (A2), each row is from the same cone type, and each column is from the same DKL hue. On the left side of each cone type, there are two color disks, which represent the ON-phase (upper) and OFF-phase (lower), respectively. The color disks on top of each row represent the hue-phase and grey-phase, and the hue direction is labeled at bottom of **c**.

c, The angles defined by the lines intersecting the regions of peak pixel densities in the upper and lower quadrants of each bivariate histogram for each cone type. X-axis is the hue direction in DKL isoluminant plane, Y-axis is the angle calculated from each bivariate histogram (see "Image Processing for COFD Contours, Histograms and ISI DKL hue tuning curves").

d, DKL hue direction map of animal A1. The inserted color key is the same as the one shown in Fig 5f. The similar map from A2 is shown in Fig. 6b-c. Scale bar: 1mm.

e, The hue tuning curves calculated from the mean of pixel values within each COFD region on hue preference maps in animal A1.

f, The hue tuning curves calculated from the mean of pixel values within each COFD-overlapping region on hue preference maps in animal A1.

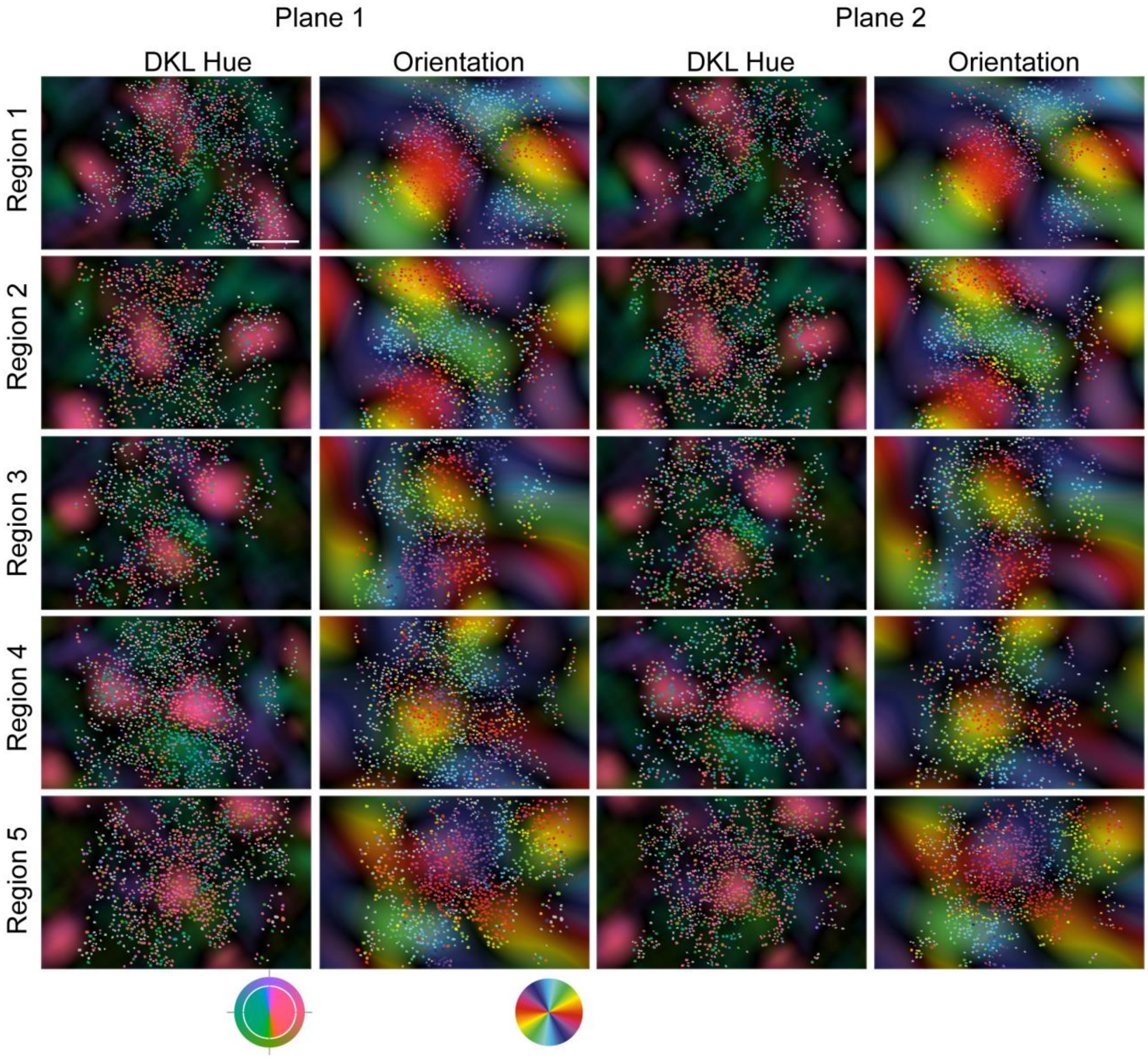

Supplementary Fig. 5 More cases of DKL hue preference maps and orientation maps.

Each row is one imaging region with 2 planes. All 5 regions, as the same 5 regions shown in Supplementary Fig. 1, are from A1. Each image has 2PCI image overlays on top of the polar map calculated from ISI images. Grey dot in 2PCI image indicates a neuron is not significantly tuned by DKL hue or orientation. Same to Fig. 3J, DKL hue preference maps of 2PCI and ISI are plotted using the DKL hue shown as the outer ring of the color key at bottom. The actual color used for stimulation is shown as the inner disk of the color key. Details of stimuli are shown in Methods. For orientation map of 2PCI and ISI, the orientation is indicated by the angle (0 to 180 degree) of color in the color key at bottom of the orientation map. We used the alignment of 2PCI orientation map and ISI orientation polar map as the secondary check about alignment between 2PCI and ISI maps based on surface blood vessel maps. Scale bar: 200 μm, applies to all images.

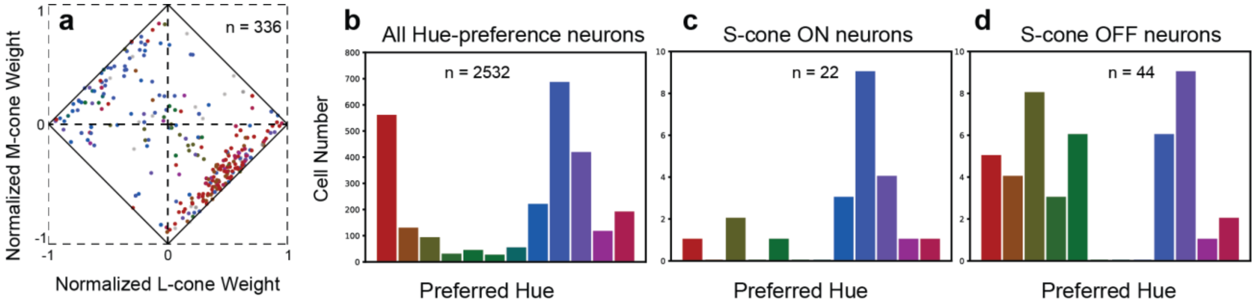

Supplementary Fig. 6 CIE hue preference and its relationship with Cone weights.

a, Relationships between CIE hue preferences and cone weights calculated from STAs. 336 neurons with significant L, M, or S-cone STAs from 4 imaging regions (3 from A3 and 1 from A4, 6 planes total) are plotted. Each neuron in the plot is colored according to its preferred CIE hue. Neurons with significant STAs, but which are not hue selective, are plotted as grey.

b-d, The distribution of preferred CIE hues of all hue-selective neurons (**b**), hue-selective neurons with significant S-ON STA (**c**), and hue-selective neurons with significant S-OFF STA (**d**). The preferred CIE hue is shown as the color of bar, and the number of neurons sampled is shown in each panel.

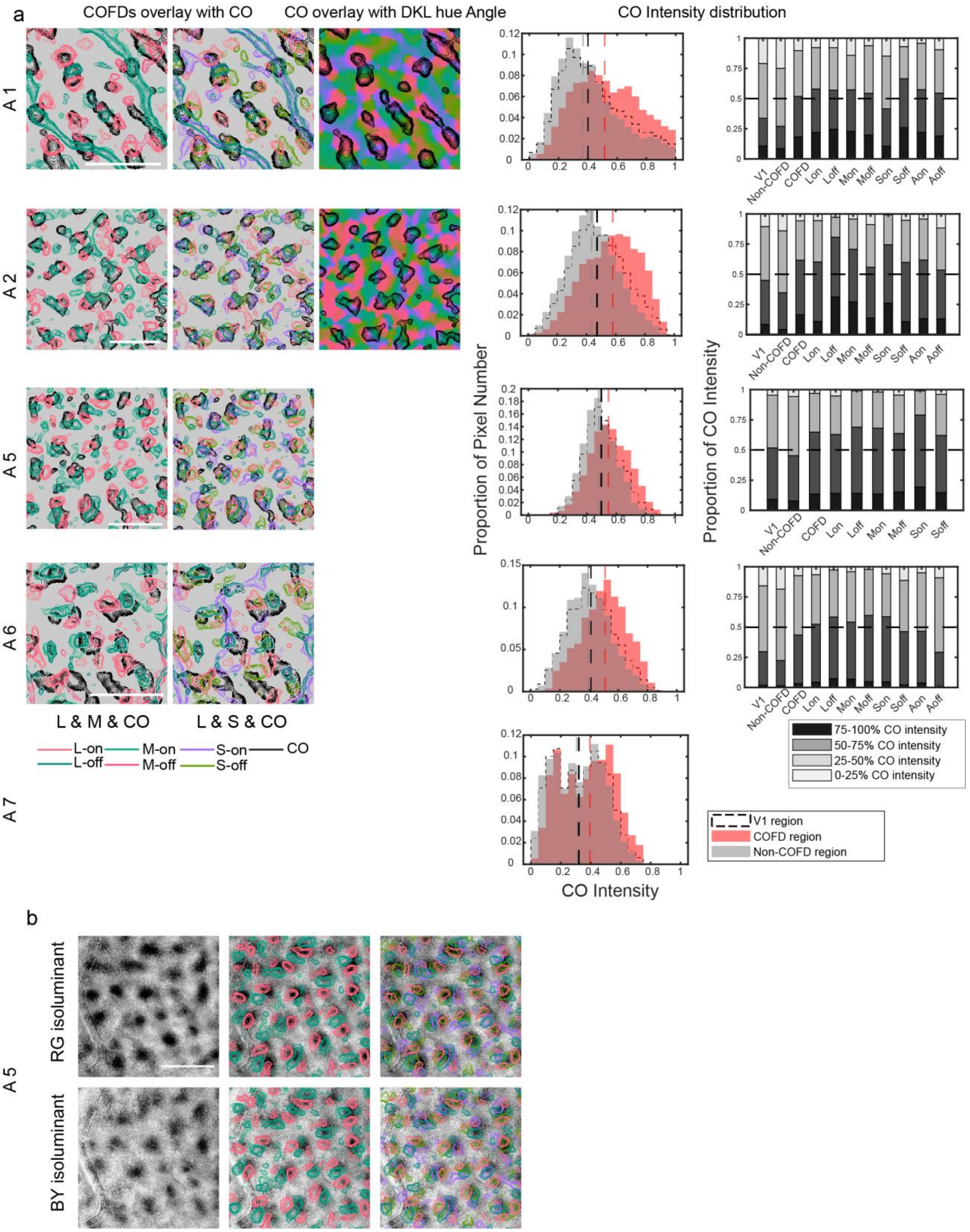

a
COFDs overlay with CO
CO overlay with DKL hue Angle
CO Intensity distribution
A 1
A 2
A 5
A 6
A 7
L & M & CO
L & S & CO
L-on
L-off
M-on
M-off
S-on
S-off
CO
Proportion of Pixel Number
CO Intensity
Proportion of CO Intensity
V1
Non-COFD
COFD
Lon
Loff
Mon
Moff
Son
Soff
Aon
Aoff
75-100% CO intensity
50-75% CO intensity
25-50% CO intensity
0-25% CO intensity
V1 region
COFD region
Non-COFD region
b
RG isoluminant
BY isoluminant

Supplementary Fig. 7 The spatial relationship of COFDs with CO blobs, DKL hue direction map, Red/Green and Blue/Yellow isoluminant color maps.

a, Each row shows results from one animal. All 5 cases are shown in this Figure, with A5 also shown in Fig. 7. First column, L- and M-COFD contours overlay with CO blob contours. Second column, L- and S-COFD contours overlay with CO blob contours. Third column, which only consists of two cases (A1 and A2), is CO blob contours overlay on top of DKL hue direction map. Fourth column is the histograms of CO intensity distributions within COFD, outside COFD (Non-COFD) and whole V1 regions. Dash lines are median of the distribution in each region. In all cases, the CO intensity distribution is biased in COFD regions vs. Non-COFD regions, and the difference is statistically significant (Wilcoxon signed-rank test, two-sided). Fifth column shows more comparison of the CO intensity distribution, including each COFD sub-region, achromatic ON (Aon) and OFF regions (Aoff). Note that because we did not identify the ON/OFF phase in A7, only comparison between COFD and Non-COFD was made. Scale bar for each case: 1 mm.

b, COFD contours overlay on top of the Red/Green and Blue/Yellow isoluminant color maps. Note that these maps were from the same region of A5 in **a** and Fig. 7e,f. Scale bar for each case: 1 mm.

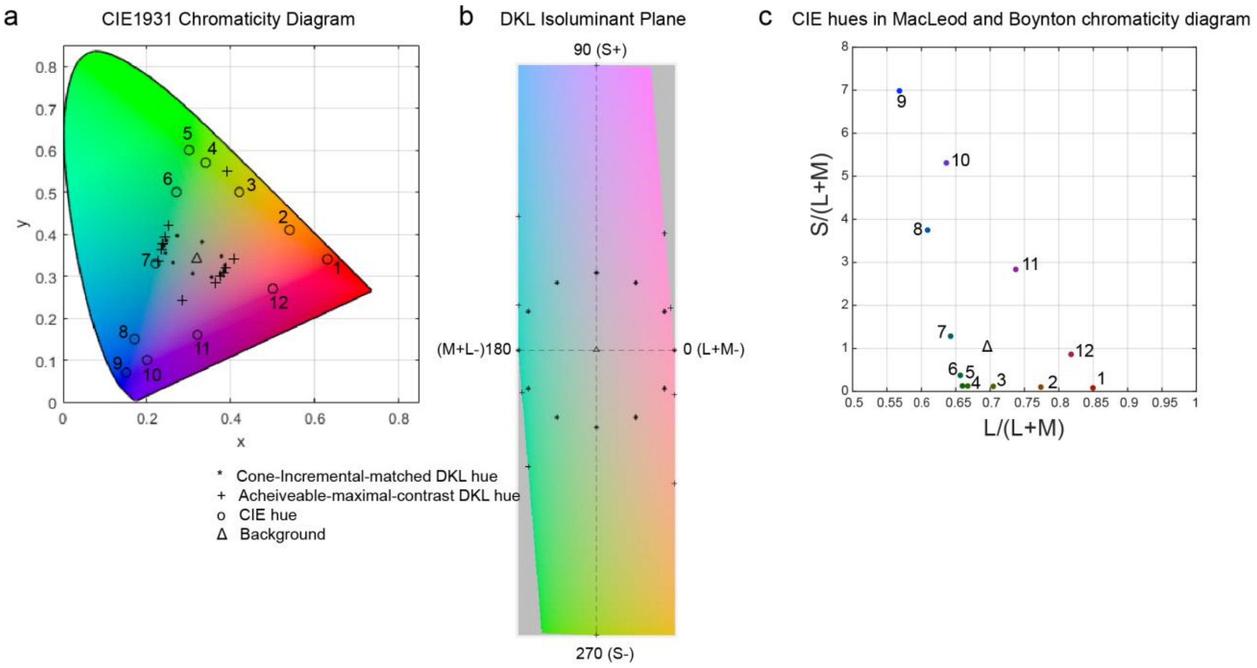

Supplementary Fig. 8 DKL and CIE hues applied in this research.

a, Cone-increment-matched DKL hue (12 hues used in A1), achievable maximal contrast DKL hue (12 hues used for A2), and CIE hues (12 hues used in A3 and A4) are shown in CIE 1931 chromaticity diagram. Twelve CIE hues were labeled with numbers (1-12) to refer the same hue replotted in **c**.

b, Cone-increment-matched DKL hue and achievable maximal contrast DKL hue are shown DKL isoluminant plane.

c, Twelve CIE hues were replotted in Macleod and Boynton chromaticity diagram.

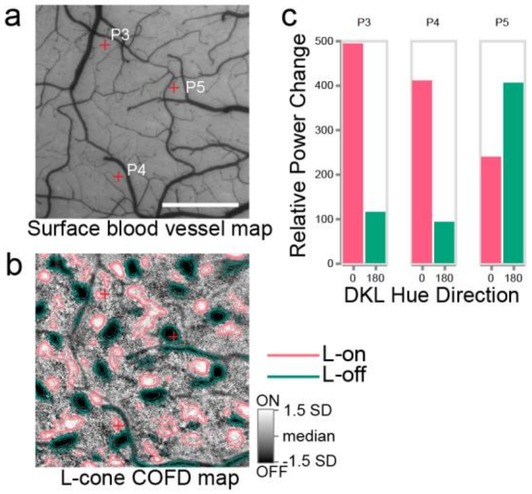

Supplementary Fig. 9 L-cone COFD map of animal A2 and electrophysiological recordings in COFDs

a-b, Surface blood vessel map (**a**) and L-cone COFD map (**b**) for guiding electrode penetration within COFDs. Three penetrations (red-cross) were shown in (**a-b**). Scale bar in **a**: 1 mm, applies to **a-b**.

c, The relative change of LPF power histograms from three penetrations shows the dominant response to DKL hue at 0 degree (P3 and P4) or 180 degree (P5).

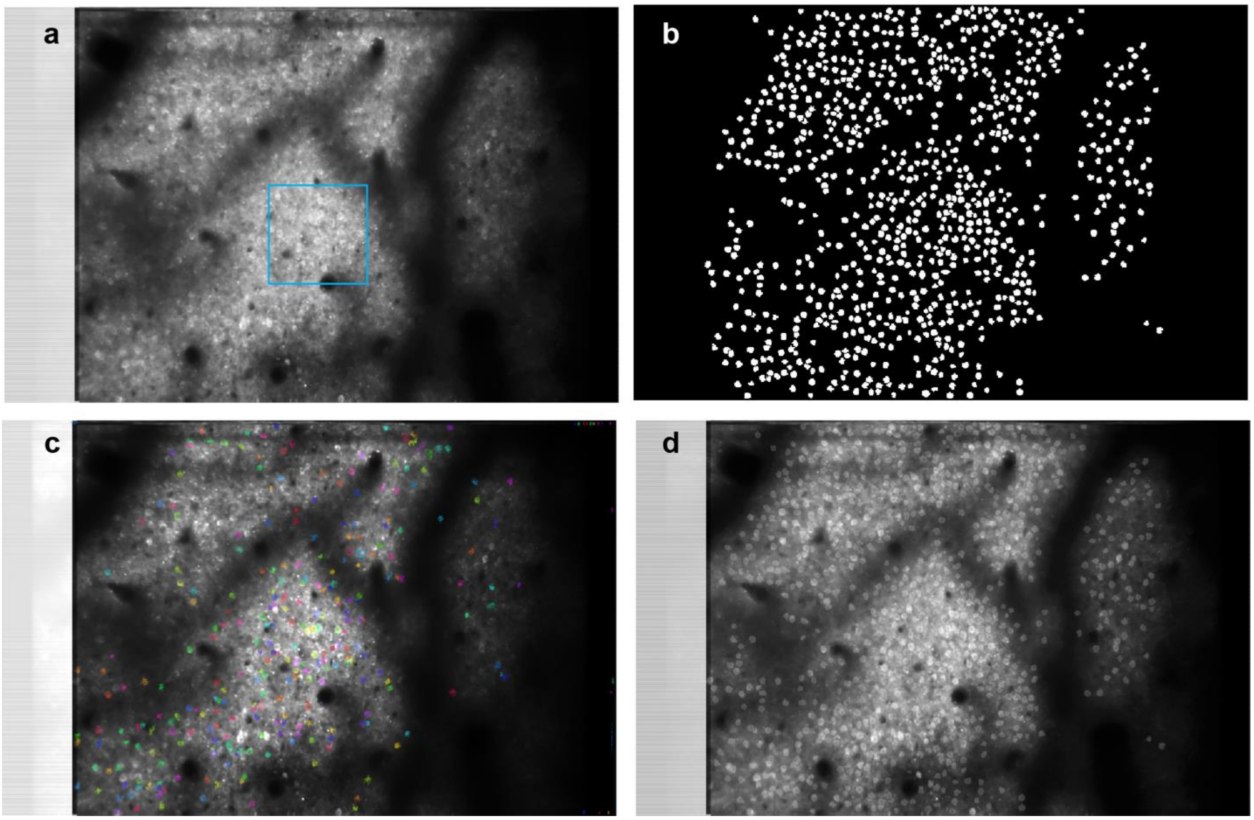

Supplementary Fig. 10 Comparison of our manually selected ROIs with ROIs generated by Suite2p as requested by reviewer.

a, The same imaging plane shown in Fig. 2d; Fig. 2d is the region shown in the square.

b, Manually selected ROIs from image (**a**).

c, Suite2p generated ROIs overlaid on image (**a**)

d, Manually selected ROIs overlaid on image (**a**)

Supplementary Table 1. Case summary from seven animals reported in this research.

Animal ID	Contributions to Fig.	ISI Results	2PCI Results	2PCI Planes	Ephys	CO
A1	2, 3, 5, Supplementary 1, 2, 4, 5, and 7	COFD & DKL hue-phase maps, Orientation map	Cone and achromatic ON/OFF map, DKL Hue map, Cone weight distribution, Orientation map	5 regions (10 planes)	None	Yes
A2	4, 5, 6, Supplementary 3, 4, 7, and 9	COFD & DKL hue-phase maps, Orientation map	None		Yes	Yes
A3	Supplementary 1 and 6	None	Cone and achromatic ON/OFF map, CIE Hue map, Cone weight distribution	3 regions (5 planes)	None	None
A4	Supplementary 1 and 6	None	Cone and achromatic ON/OFF map, CIE Hue map, Cone weight distribution	1 region (1 plane)	None	None
A5	7, Supplementary 2 and 7	COFD maps, Red/Green and Blue/Yellow color maps	None		Yes	Yes
A6	Supplementary 2 and 7	COFD maps	None		None	Yes
A7	Supplementary 2 and 7	COFD maps	None		None	Yes

Supplementary Table 2. CIE coordinates of presented hues.

CIE Hue				Cone-increment-matched DKL Hue				Achievable-maximal-contrast DKL Hue			
Hue Name	CIE Coordinates			Hue Direction (degree)	CIE Coordinates			Hue Direction (degree)	CIE Coordinates		
	x	*y*	*Y* (cd/m²)		*x*	*y*	*Y* (cd/m²)		*x*	*y*	*Y* (cd/m²)
Red	0.63	0.34	10.03	0	0.36	0.29	71.54	0	0.38	0.31	85.90
Orange	0.54	0.41	10.03	30	0.36	0.29	70.99	30	0.38	0.30	85.78
Yellow	0.42	0.50	10.00	60	0.34	0.27	69.92	60	0.36	0.28	85.13
Lime	0.34	0.57	9.91	90	0.29	0.28	70.06	90	0.28	0.24	82.61
Green	0.30	0.60	9.89	120	0.24	0.31	70.07	120	0.23	0.34	84.74
Green	0.27	0.50	10.23	150	0.22	0.33	70.43	150	0.23	0.36	85.06
Cyan	0.22	0.33	10.01	180	0.22	0.35	70.39	180	0.24	0.38	85.46
Blue	0.17	0.15	9.96	210	0.23	0.36	70.85	210	0.24	0.39	85.64
Blue	0.15	0.07	10.08	240	0.25	0.37	71.51	240	0.25	0.42	86.57
Purple	0.20	0.10	9.97	270	0.31	0.35	71.83	270	0.39	0.55	89.11
Magenta	0.32	0.16	9.96	300	0.36	0.32	71.84	300	0.41	0.34	87.19
Red	0.50	0.27	9.99	330	0.37	0.30	71.49	330	0.39	0.32	86.63
Background	0.33	0.33	10.01	Background	0.30	0.31	71.46	Background	0.32	0.34	86.29
