# Supplementary material for: Cone opponent functional domains in primary visual cortex combine signals for color appearance mechanisms
Source: Nat Commun. 2022 Oct 25;13:6344. doi: 10.1038/s41467-022-34020-2 (PMC9596481; doi:10.1038/s41467-022-34020-2)
Supplement: Supplementary file 2 — Reporting Summary [file 41467_2022_34020_MOESM2_ESM.pdf]

Corresponding author(s): Edward M. Callaway
Last updated by author(s): Aug 21, 2022

Reporting Summary

Nature Portfolio wishes to improve the reproducibility of the work that we publish. This form provides structure for consistency and transparency in reporting. For further information on Nature Portfolio policies, see our Editorial Policies and the Editorial Policy Checklist.

Statistics

For all statistical analyses, confirm that the following items are present in the figure legend, table legend, main text, or Methods section.

n/a	Confirmed	
☐	☒	The exact sample size (*n*) for each experimental group/condition, given as a discrete number and unit of measurement
☐	☒	A statement on whether measurements were taken from distinct samples or whether the same sample was measured repeatedly
☐	☒	The statistical test(s) used AND whether they are one- or two-sided<br>*Only common tests should be described solely by name; describe more complex techniques in the Methods section.*
☒	☐	A description of all covariates tested
☐	☒	A description of any assumptions or corrections, such as tests of normality and adjustment for multiple comparisons
☐	☒	A full description of the statistical parameters including central tendency (e.g. means) or other basic estimates (e.g. regression coefficient) AND variation (e.g. standard deviation) or associated estimates of uncertainty (e.g. confidence intervals)
☐	☒	For null hypothesis testing, the test statistic (e.g. *F*, *t*, *r*) with confidence intervals, effect sizes, degrees of freedom and *P* value noted<br>*Give P values as exact values whenever suitable.*
☒	☐	For Bayesian analysis, information on the choice of priors and Markov chain Monte Carlo settings
☒	☐	For hierarchical and complex designs, identification of the appropriate level for tests and full reporting of outcomes
☐	☒	Estimates of effect sizes (e.g. Cohen's *d*, Pearson's *r*), indicating how they were calculated

Our web collection on statistics for biologists contains articles on many of the points above.

Software and code

Policy information about availability of computer code

Data collection	Matlab (R2014, R2017, and R2019, Mathworks Inc., MA);<br>ISI imager (customized Matlab code)<br>Scanbox (customized version, Neurolabware, Los Angeles, CA);<br>SpikeGLX v3.0 (http://billkarsh.github.io/SpikeGLX);<br>Psychophysics Toolbox Version 3;
Data analysis	Scanbox (https://scanbox.org/)<br>Adobe Photoshop (v22.x)<br>ImageJ (https://imagej.nih.gov/ij/)<br>Customized Matlab code (R2019, Mathworks Inc., MA) has been uploaded in Github. Link is provided in the main text.<br>Customized Julia code (v1.3-1.5, https://julialang.org/) has been uploaded in Github. Link is provided in the main text.

For manuscripts utilizing custom algorithms or software that are central to the research but not yet described in published literature, software must be made available to editors and reviewers. We strongly encourage code deposition in a community repository (e.g. GitHub). See the Nature Portfolio guidelines for submitting code & software for further information.

Data

Policy information about availability of data

All manuscripts must include a data availability statement. This statement should provide the following information, where applicable:

- Accession codes, unique identifiers, or web links for publicly available datasets
- A description of any restrictions on data availability
- For clinical datasets or third party data, please ensure that the statement adheres to our policy

All data necessary to support the paper's conclusions are present in the main text, supplemental information, or available from the corresponding author upon reasonable request. The results presented here are based on more than 5TB of raw data and include data measuring activity during stimulus conditions that are not relevant to the present manuscript. The authors are presently conducting further analyses of that data which will be published at a later time. These data are not structured to allow data relevant to this manuscript to be separated from that which is reserved for future publication.

Field-specific reporting

Please select the one below that is the best fit for your research. If you are not sure, read the appropriate sections before making your selection.

☒ Life sciences ☐ Behavioural & social sciences ☐ Ecological, evolutionary & environmental sciences

For a reference copy of the document with all sections, see nature.com/documents/nr-reporting-summary-flat.pdf

Life sciences study design

All studies must disclose on these points even when the disclosure is negative.

Sample size	Data were collected from 7 animals, five (5 hemispheres)of which contributed to the ISI data, and three (9 regions, 13117 visually responsive neurons) contributed to the 2PCI data. We did not predetermine the sample size. It is readily apparent from the provided analysis of the data set that there would be no change to any conclusion with the addition of data from more animals. It would not be justified to sacrifice more non-human primates to increase the size of this data set.
Data exclusions	For 2PCI, neurons that are not visually responsive were excluded from further analysis. Neurons that are not significantly tuned by hue or orientation were excluded for plotting hue or orientation preference maps (Fig. 5 and Extended Data Fig. 5).<br>For Spike-triggered Average and Cone Weight Calculation analysis, neurons without significant STA (see the three criteria in the Method) were excluded for plotting ON/OFF-dominant receptive maps (Figs. 2, 3 and Extended Data Fig. 1) and cone weight calculation (Fig. 5, Extended Data Fig. 6).<br>For ISI image analysis, pixels outside response masks are excluded for quantitative analysis (Figs. 4, 5, 6, Extended Data Figs. 2, 3, 4).<br>For CO image analysis, regions that are damaged by electrode penetrations are excluded from quantitative analysis due to the loss of CO staining (Fig. 7).
Replication	We successfully verified ISI data from five animals (5 hemispheres), and verified 2PCI data from two animals (9 brain regions).
Randomization	This study aimed to test whether a neuron/a brain region responds differently to different visual stimuli. We did not compare between different neurons/regions, so we did not randomize neurons/regions. For continuous-periodic ISI experiments, visual stimuli were presented periodically for Fourier analysis. Except for this, other visual stimuli (episodic) were presented randomly.
Blinding	The investigators were not blinded to group allocation during data collection and analysis in this study. We aimed to replicate the same results from different anesthetized animals using the same visual stimulus set. Because only normal animals were used, no blinding was needed.

Reporting for specific materials, systems and methods

We require information from authors about some types of materials, experimental systems and methods used in many studies. Here, indicate whether each material, system or method listed is relevant to your study. If you are not sure if a list item applies to your research, read the appropriate section before selecting a response.

Materials & experimental systems

n/a	Involved in the study	
☒	☐	Antibodies
☒	☐	Eukaryotic cell lines
☒	☐	Palaeontology and archaeology
☐	☒	Animals and other organisms
☒	☐	Human research participants
☒	☐	Clinical data
☒	☐	Dual use research of concern

Methods

n/a	Involved in the study	
☒	☐	ChIP-seq
☒	☐	Flow cytometry
☒	☐	MRI-based neuroimaging

Animals and other organisms

Policy information about studies involving animals; ARRIVE guidelines recommended for reporting animal research

Laboratory animals	Macaque monkeys (M. fascicularis); Four Males and Three Females; Adult (7-13 years old)
Wild animals	This study did not involve wild animals.
Field-collected samples	This study did not involve samples collected from the field.
Ethics oversight	All procedures involving live animals were conducted in accordance with the guidelines of the NIH and were approved by the Institutional Animal Care and Use Committee (IACUC) at The Salk Institute for Biological Studies.

Note that full information on the approval of the study protocol must also be provided in the manuscript.
